# A *Drosophila melanogaster* model for *TMEM43*-related arrhythmogenic right ventricular cardiomyopathy type 5

**DOI:** 10.1007/s00018-022-04458-0

**Published:** 2022-07-22

**Authors:** Nora Klinke, Heiko Meyer, Sandra Ratnavadivel, Marcel Reinhardt, Jürgen J. Heinisch, Anders Malmendal, Hendrik Milting, Achim Paululat

**Affiliations:** 1 Faculty of Biology and Chemistry, Zoology and Developmental Biology, University of Osnabrück, Barbarastraße 11, 49076 Osnabrück, Germany; 2 Faculty of Biology and Chemistry, Genetics, University of Osnabrück, Barbarastraße 11, 49076 Osnabrück, Germany; 3 Herz-und Diabeteszentrum NRW, Universitätsklinik der Ruhr Universität Bochum, Erich und Hanna Klessmann-Institut für Kardiovaskuläre Forschung und Entwicklung, Georgstr. 11, 32545 Bad Oeynhausen, Germany; 4 Department of Science and Environment, Roskilde University, Universitetsvej 1, 4000 Roskilde, Denmark; 5 Center of Cellular Nanoanalytics Osnabrück (CellNanOs), Osnabrück University, Osnabrück, Germany

**Keywords:** *Drosophila*, TMEM43, LUMA

## Abstract

Arrhythmogenic right ventricular cardiomyopathy (ARVC) is a severe cardiac disease that leads to heart failure or sudden cardiac death (SCD). For the pathogenesis of ARVC, various mutations in at least eight different genes have been identified. A rare form of ARVC is associated with the mutation *TMEM43* p.S358L, which is a fully penetrant variant in male carriers. TMEM43 p.S358 is homologous to CG8111 p.S333 in *Drosophila melanogaster*. We established CRISPR/Cas9-mediated *CG8111* knock-out mutants in *Drosophila*, as well as transgenic fly lines carrying an overexpression construct of the CG8111 p.S333L substitution. Knock-out flies developed normally, whereas the overexpression of CG8111 p.S333L caused growth defects, loss of body weight, cardiac arrhythmias, and premature death. An evaluation of a series of model mutants that replaced S333 by selected amino acids proved that the conserved serine is critical for the physiological function of CG8111. Metabolomic and proteomic analyses revealed that the S333 in CG8111 is essential to proper energy homeostasis and lipid metabolism in the fly. Of note, metabolic impairments were also found in the murine *Tmem43* disease model, and fibrofatty replacement is a hallmark of human ARVC5. These findings contribute to a more comprehensive understanding of the molecular functions of CG8111 in *Drosophila*, and can represent a valuable basis to assess the aetiology of the human *TMEM43* p.S358L variant in more detail.

**Supplementary Information:**

The online version contains supplementary material available at 10.1007/s00018-022-04458-0.

## Introduction

Arrhythmogenic right ventricular cardiomyopathy (ARVC) is, in most cases, a genetic disorder with fibrofatty replacement of cardiomyocytes that is clinically characterised by ventricular tachycardia, heart failure, or sudden cardiac death (SCD). The disease may affect both ventricles and is also referred to in the literature as arrhythmogenic cardiomyopathy (ACM). The worldwide prevalence of ARVC in the population is about 1:2000 [1], with a high risk for disease onset in young men [2, 3]. ARVC is often caused by variants in genes, encoding proteins in the cardiac desmosome, including plakophilin-2 (*PKP2*), desmoglein-2 (*DSG2*), desmocollin-2 (*DSC2*), desmoplakin (*DSP*) and plakoglobin (*JUP*). Truncating variants of *PKP2* belong to the most frequent genotypes related to ARVC. All these proteins are critical to the mechanical integrity of the myocardial desmosome.

The ARVC type 5 is a rare subtype related to the gene *TMEM43* on chromosome 3p25. *TMEM43* codes for the protein TMEM43, also called LUMA, which was first identified in a proteome study on nuclear rim proteins [4]. Bengtsson et al., predicted four transmembrane domains in TMEM43 and an acidic loop with a domain of unknown function (DUF1625) located within the ER lumen. The pathogenic variant p.S358L (c.1073C>T) in *TMEM43* was first identified in the Newfoundland population [5]. The pathogenic Newfoundland haplotype turned out to represent an old European founder mutation [6]. Of note, *TMEM43* p.S358L was later identified in Spanish families with a different haplotype but comparable clinical phenotype, which supports the malignancy of this heterozygous missense variant [7]. *TMEM43* p.S358L is fully penetrant in male carriers, and more than 50% of male carriers receive heart transplantation (HTx), are affected by cardiomyopathy, or die before the age of 40 years [5–11]. Interestingly, female carriers reveal a milder cardiac phenotype for unknown reasons. Preventive implantation of an implantable cardioverter defibrillator (ICD) is practised, especially in genotype-positive males, as fatal arrhythmic events are difficult to predict [12]. Patients with a severe course of the disease can ultimately only be treated by orthotopic HTx.

Until now, only one murine knock-out (ko) model for *Tmem43* has been published [13]. However, a cardiac phenotype was only observed when the human or the murine mutant protein was overexpressed [11, 14, 15]. A recent transcriptome analysis in mice suggested that the cardiac lipid metabolism is altered by *Tmem43* p.S358L [15]. However, the precise molecular mechanism of TMEM43 and the pathomechanisms of the variant p.S358L remained unclear.

TMEM43 is a highly conserved protein with homologous domains even in bacteria and *Drosophila* [16]. This suggests that TMEM43 may play a fundamental biological role in phylogenetically distant organisms as well. Considering that serine-358 in *TMEM43* is homologous to *Drosophila melanogaster CG8111* serine-333, we generated a series of transgenic *Drosophila* lines to investigate the variant *TMEM43* p.S358L in the fly model. We found that the corresponding amino acid variant *CG8111* p.S333L causes arrhythmias and premature death also in the fly. In addition, the proteome as well as the metabolome were characteristically altered in respective animals. Thus, we present here *Drosophila* as a complementing model to further investigate the molecular mechanisms leading to ARVC5.

## Results

### *Drosophila* CG8111 is a TMEM43 homologue

*Drosophila CG8111* is located on chromosome 3 at position 66A19, and it encodes a 376 amino acid protein, for which a DIOPT-based search [17] via FlyBase [18] retrieved orthologues across all model organisms, including mammals, amphibians, fish, and insects, but not baker´s or fission yeast.

**Fig. 1:**
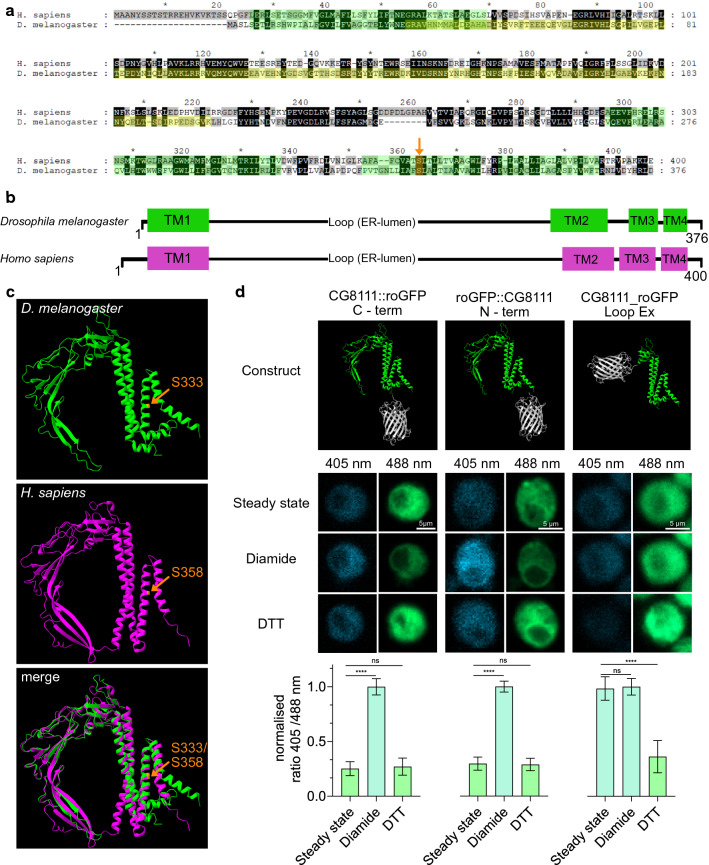
CG8111 and TMEM43 exhibit highly similar structure and topology. **a** Amino acid sequence alignment of human TMEM43 and *Drosophila* CG8111. Transmembrane domains predicted for CG8111 (based on AlphaFold) are highlighted in green; the antigen sequence, used for antibody generation is highlighted in yellow; the conserved serine-358 (human) and, serine-333 (*Drosophila*) is marked in orange (arrow). **b** Schematic overview of predicted transmembrane domains in CG8111 and TMEM43. **c** Protein structure prediction for CG8111 and TMEM43 (based on AlphaFold). **d ** Topology analysis with different roGFP-fusions of CG8111, C- (*n* = 28) and N (*n* = 12)-terminally fused roGFP faces the cytoplasm (steady state equals to DTT, reducing), roGFP inserted in exchange for the loop-domain (*n* = 32) faces the ER-lumen (steady state equals to Diamide, oxidizing). Data are shown as mean + SD. Statistical tests: depending on the normal distribution (D’Agostino–Pearson test), one-way ANOVA or Kruskal–Wallis test, followed by Dunn’s Multiple Comparison Test were performed. ****p < 0.0001

The *Drosophila* CG8111 protein exhibits approximately 34% identity with its human homologue TMEM43 (Fig. 1a). The AlphaFold algorithm [19] predicts the presence of four conserved transmembrane domains (TM), both in the *Drosophila* and the human protein, with a larger acidic loop between TM1 and TM2 (Fig. 1b, c). This supports the structural predictions of Bengtsson et al. [16]. The missense variant p.S358L in human TMEM43 [5] locates within TM3 of the protein. Serine-358 in human TMEM43 is homologous to serine-333 in *Drosophila* CG8111 and is phylogenetically conserved throughout all metazoan TMEM43-homologues known so far (Fig. 1a). The AlphaFold models predict a common structural role for the serine-358 in TMEM43 and serine-333 in CG8111 because the hydroxyl groups at these positions are required for hydrogen bond formation between helices 3 and 4 (Fig. 7b).

The topology of human TMEM43 has been explored previously by utilising epitope accessibility as well as protease-protection assays. Based on these experiments, four TMs were proposed, with the N- and C-termini of the protein facing the cytoplasmic/nucleoplasmic side. In addition, a loop of about 250 amino acids localising in the endoplasmic reticulum (ER) lumen was predicted between TM1 and TM2 [16]. To determine whether the *Drosophila* protein shares the same topological features as its human homologue, we used redox-sensitive GFP (roGFP) [20] fused to either the N- or the C-terminus of CG8111. In addition, we analysed a construct with roGFP inserted between TM1 and TM2 in a partially deleted loop domain (Fig. 1d). S2 cells were individually transfected with all constructs, and roGFP fluorescence was quantified under steady-state as well as under oxidising or reducing conditions, respectively. An evaluation of the resulting fluorescence intensity ratios confirmed that both termini face the cytoplasm/nucleoplasm, whereas the loop domain between TM1 and TM2 locates in the ER lumen (Fig. 1d). We concluded from our experiments that *Drosophila* CG8111 and human TMEM43 [16] exhibit an identical membrane topology.

### *Drosophila* CG8111 is ubiquitously expressed at all developmental stages and localises in the ER membrane and nuclear envelope

**Fig. 2:**
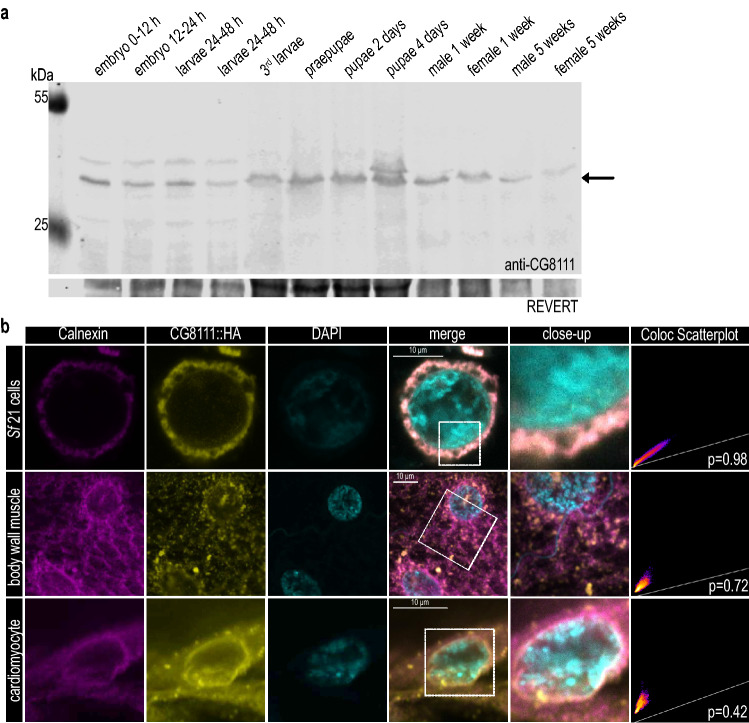
Expression and localisation of CG8111 corresponds to human TMEM43. **a** Western blot analysis indicates that CG8111 (arrow) is present throughout all developmental stages. **b** *Drosophila* CG8111 localises within the ER compartment and the nuclear membrane in *Sf*21-cells, muscle tissue, and cardiomyocytes. Co-localisation of CG8111 and Calnexin was calculated by Pearson’s correlation coefficient

**Fig. 3:**
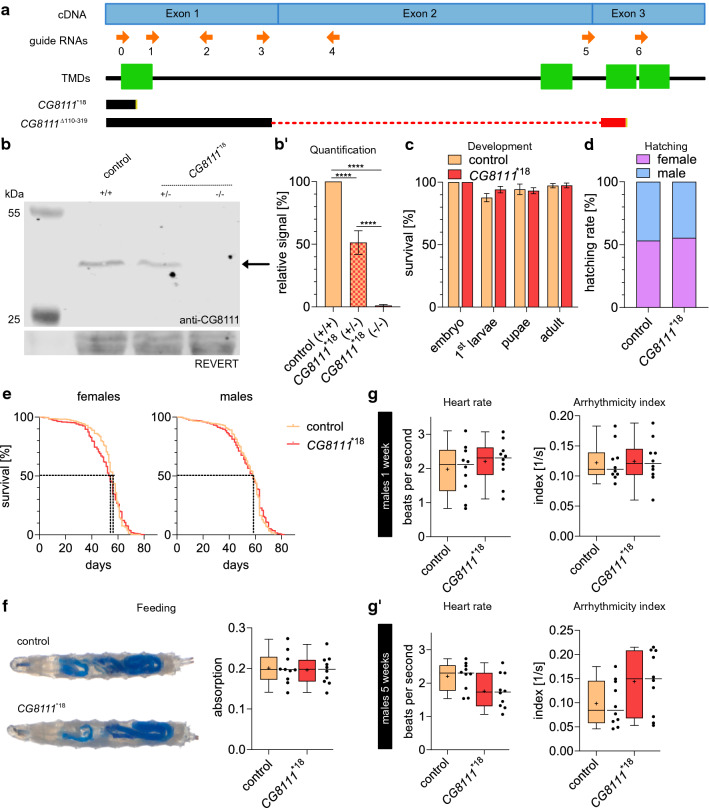
CG8111 knock-out shows no defects in development or cardiac function. **a** Schematic overview of the guide RNAs used for CRISPR-mutagenesis, predicted transmembrane domains and two successfully generated ko mutants (black bar = in-frame sequence; yellow = stop codon; red dotted line = deletion; red bar = out-of-frame sequence). **b, b’** Western blot and quantification of CG8111 signals (arrow) in heterozygous and homozygous ko mutants showed reduced signals by ~ 50 and 100%, respectively, relative to controls. **c** During development, no differences in viability were found in CG8111 ko animals (*n* = 450) compared with the control (*n* = 407). **d** The ratio of hatched female and male flies is not affected in the ko (*n* = 195) compared with the control (*n* = 235). **e** Life span of CG8111 ko flies (n (females) = 427, n (male) = 423) shows no differences compared with the control (n (females) = 383, n (males) = 429) (median life span: 54 days (females) 58 days (males)). **f** Feeding behaviour is not affected in ko third larvae. **g, g’** Heart rate and arrhythmicity index of 1- and 5-week-old male flies show no significant differences between control and ko animals. Data are shown as bars with mean, with SEM (**b’**) or SD (**c**), as Kaplan Meyer curve (**e**) or as box and whiskers (**f**, **g**, **g’**). The median is indicated by the centre line of the boxplot; upper and lower bounds indicate 75th and 25th percentiles, respectively; whiskers indicate the minimum and maximum. The average is shown by + . Statistical tests: One-way ANOVA, followed by Tukey’s multiple comparison test (**b’**), two-way ANOVA, followed by Tukey’s multiple comparison test (**c**), two-way ANOVA (**d**), Log-rank (Mantel–Cox) test (**e**, **e’**) and unpaired *t*-test (**f**, **g**, **g’**) were performed. ****p < 0.0001

To examine the expression and subcellular localisation of CG8111 in time and space, we generated an antibody against the part of the protein ranging from amino acids 34–168 (for details, see the material and methods section and Fig. 1a). The specificity of the antibody was tested by Western blot analyses using both, *Sf*21 cells, untransfected and transfected with CG8111^WT^::HA (Supplemental Fig. 1a), and with wild-type and ko mutant *Drosophila* lines (generated in this study, see below). The antibody detected a protein with an apparent molecular mass of approximately 40 kDa, which was slightly below the predicted mass of 43 kDA for CG8111. This band was absent in CRISPR/Cas9 homozygous ko mutants, thus confirming the specificity of the antibody (Fig. 3b). We used this antibody to assess the abundance of CG8111 at different developmental stages by probing protein extracts isolated from embryos, larvae, pupae, and adults (Fig. 2a). We found that CG8111 was expressed at all developmental stages. These data were in agreement with genome-wide microarray expression studies that showed that *CG8111* transcripts were present at all developmental stages in all tested tissues [21]. We conclude that CG8111 is an ubiquitously expressed protein.

Our anti-CG8111 antibody was not applicable to immunohistochemistry under the conditions we had tested so far. As an alternative approach, we established transgenic flies carrying either CG8111^WT^::HA or CG8111^p.S333L^::HA fusion constructs. Similar constructs were used to transfect *Sf*21 cells. In both assays, we used anti-HA antibodies for localisation experiments. Proper expression of the fusion constructs was confirmed by Western blot analyses (Supplemental Fig. 1a, b). In transgenic *Drosophila*, we used the UAS/GAL4 system to express the CG8111 fusion proteins specifically in cardiomyocytes and somatic muscles. CG8111::HA localised in the ER compartment in these tissues, as confirmed by substantial co-localisation with the ER chaperone protein Calnexin. Additionally, CG8111::HA was detected in the nuclear envelope (Fig. 2b). By contrast, other membranes, such as those of the intracellular trafficking pathway, or the plasma membrane, were not stained in our experiments. We conclude from our results that human TMEM43 and its *Drosophila* homologue CG8111 localise in the same subcellular membrane compartments, which are the ER as well as the nuclear envelope.

### *CG8111* knock-out mutants are viable and fertile, and exhibit normal cardiac performance upon ageing

To investigate the physiological relevance of CG8111 in vivo, we established an invertebrate global ko model in *Drosophila* using CRISPR (Clustered Regularly Interspaced Short Palindromic Repeats)-Cas9-mediated targeted mutagenesis. In total, six guide RNAs (gRNAs) were generated by in vitro transcription and injected into *vasa*-Cas9 transgenic *Drosophila* (Fig. 3a). In addition, we utilised a transgenic fly line from the “Transgenic RNAi project” (TKO) collection [22], which expresses a single sgRNA to target *CG8111* (FBrf0234795; Fig. 3a). As a result of the mutagenesis approach, we obtained different *CG8111* mutant alleles. We selected two *CG8111* alleles for detailed analyses. CG8111^*18^ harbours a small deletion that causes a frame shift with a premature opal stop at codon 18. CG8111^Δ110-319^ carries a larger deletion that eliminates most of the gene´s open reading frame (Fig. 3a). Hereafter, only data for CG8111^*18^ flies are shown, as both *Drosophila* mutants behaved similarly. A Western blot analysis of protein extracts prepared from homozygous mutants and controls confirmed loss of CG8111 in ko flies (Fig. 3b,b’). We found that homozygous *CG8111* mutant flies developed normally (Fig. 3c) and were fully viable (Fig. 3d) under laboratory breeding conditions.

Furthermore, longevity (Fig. 3e) and feeding behaviour (Fig. 3f) were investigated. In all cases, the *CG8111* ko animals behaved like the controls. Next, we analysed whether *CG8111* ko flies displayed arrhythmias or other cardiac impairments. We observed that *CG8111* ko mutants displayed normal heart function under laboratory conditions (Fig. 3g, g’). Thus, we conclude that *Drosophila* CG8111 is dispensable for the development and viability of the animal, under laboratory conditions, similar to what has been observed in ko mice before [13]. In particular, arrhythmias or other cardiac dysfunctions were not detectable in the *Drosophila* model, which was also in line with the data on the *Tmem43* ko in mice [13].

### Overexpression of CG8111^p.S333L^ causes premature death

To further explore the pathogenic genotype in the *Drosophila* system, we generated transgenic fly lines carrying constructs either with the wild-type or the mutant CG8111. In addition to the endogenous gene, the inducible UAS/GAL4 system allows the production of both protein variants in a time- and tissue-specific manner. We used untagged CG8111^WT^ and CG8111^p.S333L^ to generate the first set of transgenes that were devoid of any tag-derived artificial effect. Furthermore, the transgenic *Drosophila* lines were obtained via site-specific integration to ensure comparable expression levels of the constructs. Wild-type and mutant CG8111 were expressed at similar levels, indicating that the mutant variant p.S333L has no effect on protein stability or turnover rates (Supplemental Fig. 1b). Furthermore, CG8111^p.S333L^ localised in the same cellular compartments and exhibited the same membrane topology as the wild-type protein (Supplemental Fig. 2a, b).

**Fig. 4:**
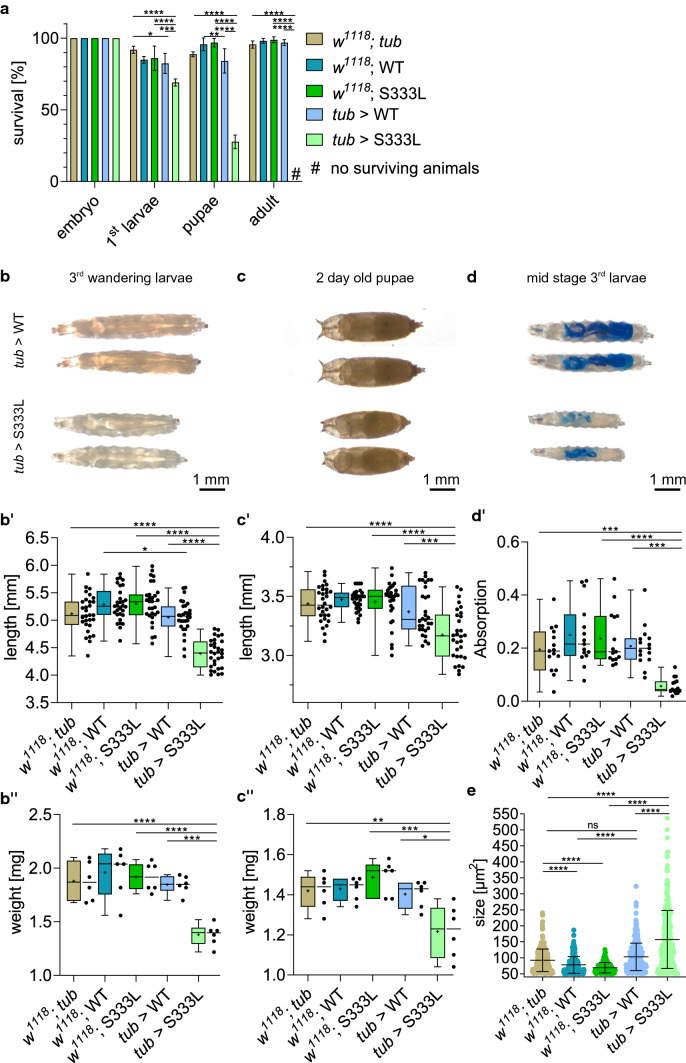
Overexpression of CG8111 p.S333L leads to lethality and growth defects. **a** Developmental assay of animals overexpressing CG8111^WT^ or CG8111^p.^^S333L^ with the ubiquitous driver *tub*-GAL4 shows a reduced number of surviving animals that overexpress the mutant form (*n* = 300) in all developmental stages compared with the overexpression of the wild-type (*n* = 300) and controls (*n* = 300, 450, 457). All animals overexpressing CG8111^p.S333L^ die during pupation. **b, b’’** Wandering third larvae that overexpress CG8111^p.^^S333L^ are significantly decreased in size and weight. **c, c’’** Two-day-old pupae that overexpress CG8111^p.^^S333L^ are significantly decreased in size and weight. **d, d’** Third larvae feed less when they overexpressed CG8111^p.^^S333L^ compared with the controls. **e** Lipid droplet size is significantly increased in wandering third larvae that overexpressed CG8111^p.^^S333L^. Data are shown as mean with SD (**a**), as box and whiskers (**b’**, **b’’**, **c’**, **c’’**, **d’**) or as scatter dot plot with mean with SD (**e**). The median is indicated by the centre line of the boxplot; upper and lower bounds indicate 75th and 25th percentiles, respectively; whiskers indicate the minimum and maximum. The average is shown by + . Statistical tests: Two-way ANOVA, followed by Tukey’s multiple comparison test (**a**), one-way ANOVA, followed by Tukey’s multiple comparison test (**b’**, **b’’**, **c’**, **c’’**) and Kruskal–Wallis test, followed by Dunn’s multiple comparison test (**d’**, **e**) were performed. *p ≤ 0.05, **p ≤ 0.01, ***p ≤ 0.001, ****p < 0.0001

The ubiquitous overexpression of CG8111^WT^, driven by *tubulin (tub)*-GAL4, resulted in healthy and fertile animals with normal morphology and viability. However, if the CG8111^p.S333L^ mutant form was overexpressed using the same driver, we observed lethality during larval and pupal stages (Fig. 4a). More than 60% of the flies (*n* = 300) with the genotype *tub*-GAL4>*UAS*-CG8111^p.S333L^ died in the second or third larval stage, and only a small percentage of specimens, about 30%, developed into pupae. However, all these pupae failed to hatch, and no adult flies appeared, indicating a complete penetrance of the mutation. This experiment demonstrated a fatal effect of the p.S333L point mutation in CG8111 if ubiquitously expressed.

Additionally, we noted that corresponding third larvae, as well as pupae were significantly smaller and lighter than the corresponding controls (Fig. 4b, c). The reduced body weight and size indicated possible deficits in food intake or utilisation. Feeding assays using Brilliant Blue-stained yeast revealed less intake in larvae that overexpressed CG8111^p.S333L^ relative to control animals (Fig. 4d, d’). To investigate whether the reduced food intake also impacted lipid storage, we performed BODIPY staining on the animals’ adipocyte tissue, which were the larval fat bodies. Interestingly, we found that overexpression of CG8111^p.S333L^ caused a significant increase in the size of lipid droplets, indicating an impaired lipid metabolism (Fig. 4e and Supplemental Fig. 3).

### Metabolic and proteomic characterisation of CG8111^WT^ and CG8111^p.S333L^ overexpressing *Drosophila* lines

The expression of CG8111^p.S333L^ had severe physiological effects that manifested in reduced growth, smaller body size, lower body weight, and lethality at later developmental stages. We speculated that such phenotypes could be caused by malfunctions in fundamental cell metabolic processes. To test this hypothesis, we ubiquitously expressed the mutant CG8111^p.S333L^ protein and collected animals directly before the lethal phase and processed them for global metabolomic and proteomic analyses, as detailed in the following.

#### Metabolome

**Fig. 5:**
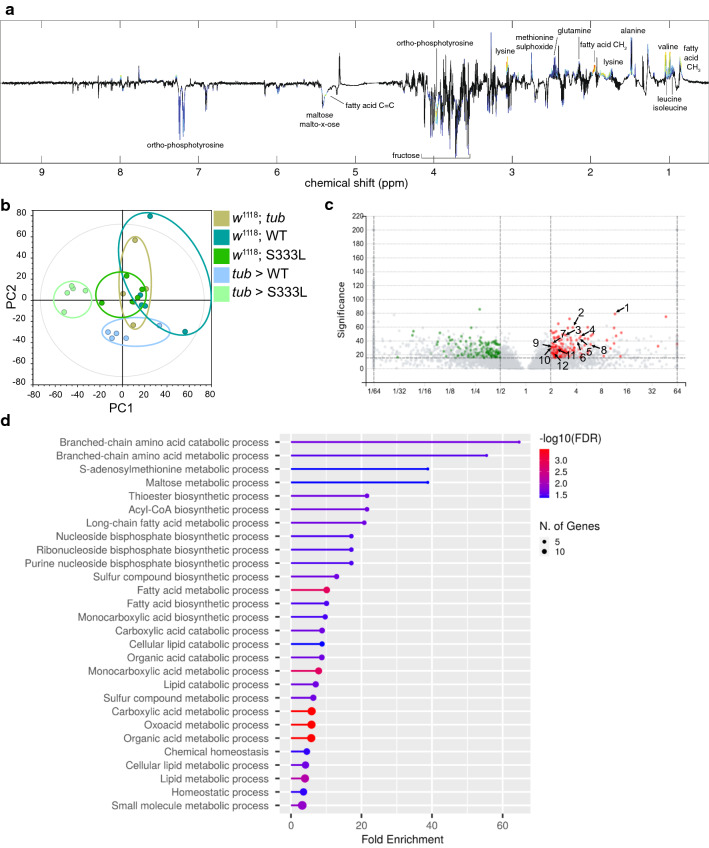
Metabolomic and proteomic analyses of *tub*>CG8111^p.S333L^ animals exhibit an impaired lipid metabolism. **a** OPLS-DA loading plot comparing animals overexpressing CG8111^p.S333L^ to all other tested genotypes. **b** PCA scores confirm that controls and the individual overexpressions (CG8111^WT^ and CG8111^p.S333L^) form separate clusters. **c** Volcano plot of proteome analysis, comparing animals overexpressing CG8111^p.S333L^ to the overexpression of CG8111^WT^ and the controls. Numbers 1–12 are the most significant proteins involved in lipid metabolism, fatty acid metabolism, and BCCA metabolism (for more detail, see Supplemental Table 1). Red = proteins more abundant in CG8111^p.S333L^ animals; green = proteins more abundant in CG8111^WT^ and control animals **d** GO analysis showing biological processes with increased abundance in animals that overexpressed CG8111^p.S333L^ compared to the overexpression of CG8111^WT^ and the controls

We extracted metabolites from animals that overexpressed either CG8111^WT^ or CG8111^p.S333L^ as well as from control animals. A principal component analysis (PCA) of NMR data confirmed the genotype-specific separation of the individual samples (controls, CG8111^WT^ and CG8111^p.S333L^) (Fig. 5b). Interestingly, fatty acids and branched-chain amino acids (BCAA) were significantly increased in CG8111^p.S333L^ overexpressing animals, relative to the CG8111^WT^ overexpressors as well as to control lines. On the other hand, fructose was significantly decreased (Fig. 5a and Supplemental Fig. 4a). Of note, increased BCAA have also been found in failing human myocardium, which is associated with the metabolic shift in heart failure.

#### Proteome

In a complementary approach, animals were collected before the lethal phase and processed for proteomic analyses. In total, more than 300 proteins were found to be differentially regulated in animals which overexpressed the mutant variant, relative to animals overexpressing wild-type CG8111 (Fig. 5c, Supplemental Fig. 4b). Interestingly, Gene Ontology (GO) analysis (ShinyGO v0.75) of proteins with increased abundance in CG8111^p.S333L^ overexpressing larvae identified BCAA (e.g. 3-hydroxyisobutyrate dehydrogenase), lipid (e.g. Lipase), and fatty acid (e.g. Acetyl-coenzyme A synthetase) metabolism as the most affected biological processes (Fig. 5d, Supplemental tab. 1) [23]. These findings further support the indication that energy and lipid metabolism are affected in animals that overexpress CG8111^p.S333L^.

### Time- and tissue-specific effects of CG8111^p.S333L^

Next, we aimed to identify the tissue as well as the developmental stage at which CG8111^p.S333L^ expression affects growth and viability most. Thus, in addition to *tub*-GAL4, ubiquitously active as well as tissue-specific GAL4 drivers were tested for their capability to induce lethality or other potential phenotypes via the expression of CG8111^p.S333L^. Among the ubiquitously active GAL4-drivers, we observed lethal effects also for *actin5C(act5C)*-GAL4. Interestingly, more than 95% of male flies with the genotype *act5C*-GAL4>*UAS*-CG8111^p.S333L^ died during development, whereas females hatched normally. Control animals of both sexes with the genotype *act5C*-GAL4>*UAS*-CG8111^WT^ developed into adulthood without defects. A reduced number of hatched males was also found when *daughterless (da)*-GAL4 was used as a driver (Table 1). However, all three driver lines were described and used as ubiquitously active drivers.

**Table 1:**
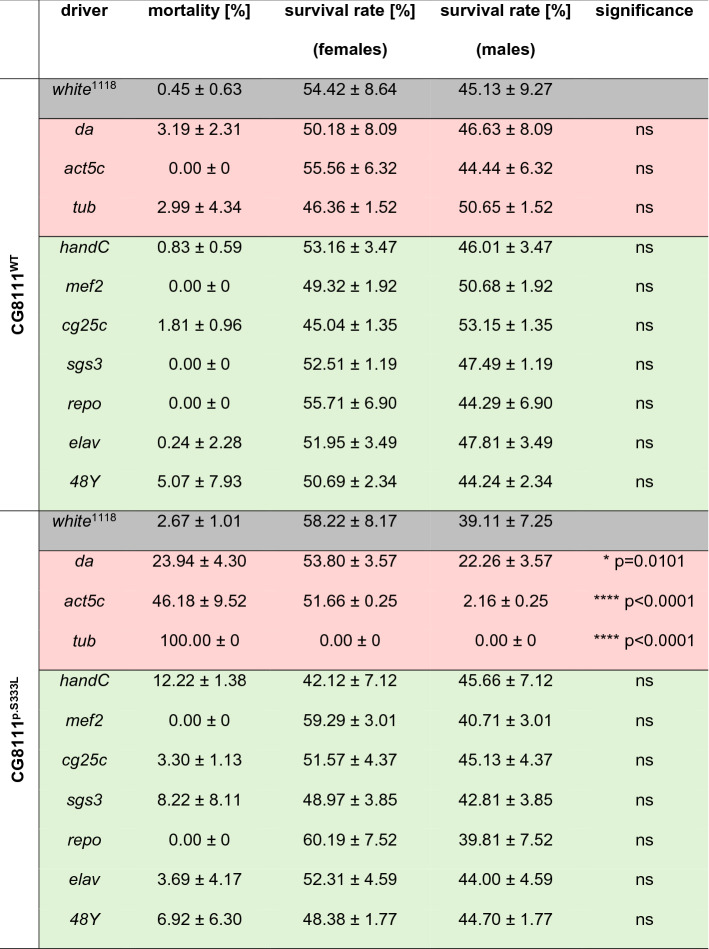
Mortality and survival rates of flies that overexpressed CG8111^WT^ or CG8111^p.^^S333L^ with different ubiquitous (red) and tissue-specific (green) drivers

Statistical tests: One-way ANOVA, followed by Tukey’s multiple comparison test

We next analysed the consequences of strong expression of CG8111^p.S333L^ in defined cell types or tissues to identify either the temporal or the spatial region when or where the organism is most sensitive to CG8111^p.S333L^. We tested *myocyte-enhancer-factor2* (*mef2*)-GAL4, which is active in cardiac, visceral, and somatic muscles during all developmental stages; *handC*-GAL4, (cardiac muscles, nephrocytes, and circular visceral muscles, all developmental stages); *reversed polarity* (*repo)*-GAL4 (glial cells of the nervous system, all developmental stages); *embryonic lethal abnormal vision* (*elav)*-GAL4 (neuronal tissue during early development and late in all neurons); *salivary gland secretion 3* (*sgs3)*-GAL4 (salivary gland cells, larval stage); *collagen-type-IV-alpha-1* (*cg25c)*-GAL4 (haemocytes and adipocytes, all developmental stages) and *48Y*-GAL4 (midgut, embryonic stage). In total, we analysed seven different GAL4 driver lines (Table 1). The expression of CG8111^p.S333L^ in distinct tissues, mediated by these tissue-specific GAL4 drivers, did not result in larval or pupal lethality in any case tested. Only ubiquitous expression of CG8111^p.S333L^ resulted in abrogation of development during the larval or pupal stage. From our experiments, we conclude that CG8111^p.S333L^ likely impairs basic cellular metabolic functions at the systemic level.

### Overexpression of mutant CG8111^p.S333L^ but not CG8111^WT^ protein causes heart arrhythmia

**Fig. 6:**
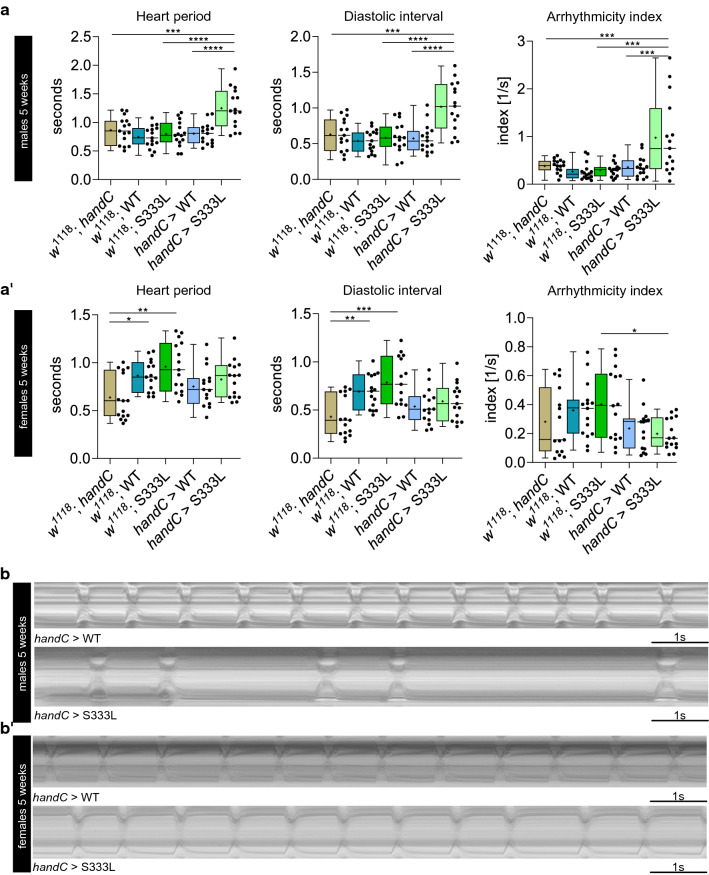
Heart parameters of *tub*>CG8111^p.S333L^ males show abnormal heartbeat. **a, a’** Heart period, diastolic interval, and arrhythmicity index of five-week-old males and females reveal that males overexpressing CG8111^p.S333L^ exhibit heart arrhythmias, whereas females are not affected. **b, b’** m-Modes of five-week-old males and females that overexpress CG8111^WT^ and CG8111^p.S333L^ are shown. Several males that overexpress the mutant protein show irregular heartbeats with long periods of the diastolic arrest. Data are shown as box and whiskers. The median is indicated by the centre line of the boxplot; the upper and lower bounds indicate 75th and 25th percentiles, respectively; whiskers indicate the minimum and maximum. The average is shown by + . Statistical tests: one-way ANOVA, followed by Tukey’s multiple comparison test. *p ≤ 0.05, **p ≤ 0.01, ***p ≤ 0.001, ****p < 0.0001

Considering the detrimental effects of TMEM43 p.S358L in human hearts, we investigated whether cardiac-specific expression of the *Drosophila* homologue CG8111^p.S333L^ also affects cardiac function in flies. Therefore, we overexpressed CG8111^WT^ and CG8111^p.S333L^ by the heart-specific driver *handC*-GAL4. In these flies, characteristic heart parameters, including the heart rate, systolic and diastolic interval, fractional shortening, and rhythmicity were measured. The expression of CG8111^p.S333L^, but not CG8111^WT^, in five-week-old males led to cardiac arrhythmias (Fig. 6a, b). We observed heart arrests for several seconds in affected animals (Supplemental video 1). Heart rate, systolic diameter, and fractional shortening were not affected. Of note, we observed arrhythmias and heart arrest only in males, whereas females displayed a normal heart performance with no signs of abnormality (Fig. 6a’, b’).

Interestingly, sex-specific differences did not only affect cardiac performance but also in terms of lethality, depending on which ubiquitous GAL4 driver was used. As depicted above (Table 1), we identified a sex-specific lethality phenotype when the mutant protein was overexpressed in all cells with the *act5c*-GAL4 driver: a lethal effect was observed in 95% of male adult flies, whereas no premature death occurred among transgenic female animals. However, 100% lethality was observed in both sexes when the *tub*-GAL4 driver was used. Based on these data, it appears conceivable that female flies are more resistant to the presence of the mutant CG8111^p.S333L^ form, compared to male flies. In line with this idea, we observed heart arrhythmias only in males when the CG8111^p.S333L^ mutant was expressed in heart tissue. However, the physiological basis for these differences remains unclear up to now.

### Relevance of serine at position p.358 in TMEM43 or p.333 in CG8111, respectively

**Fig. 7:**
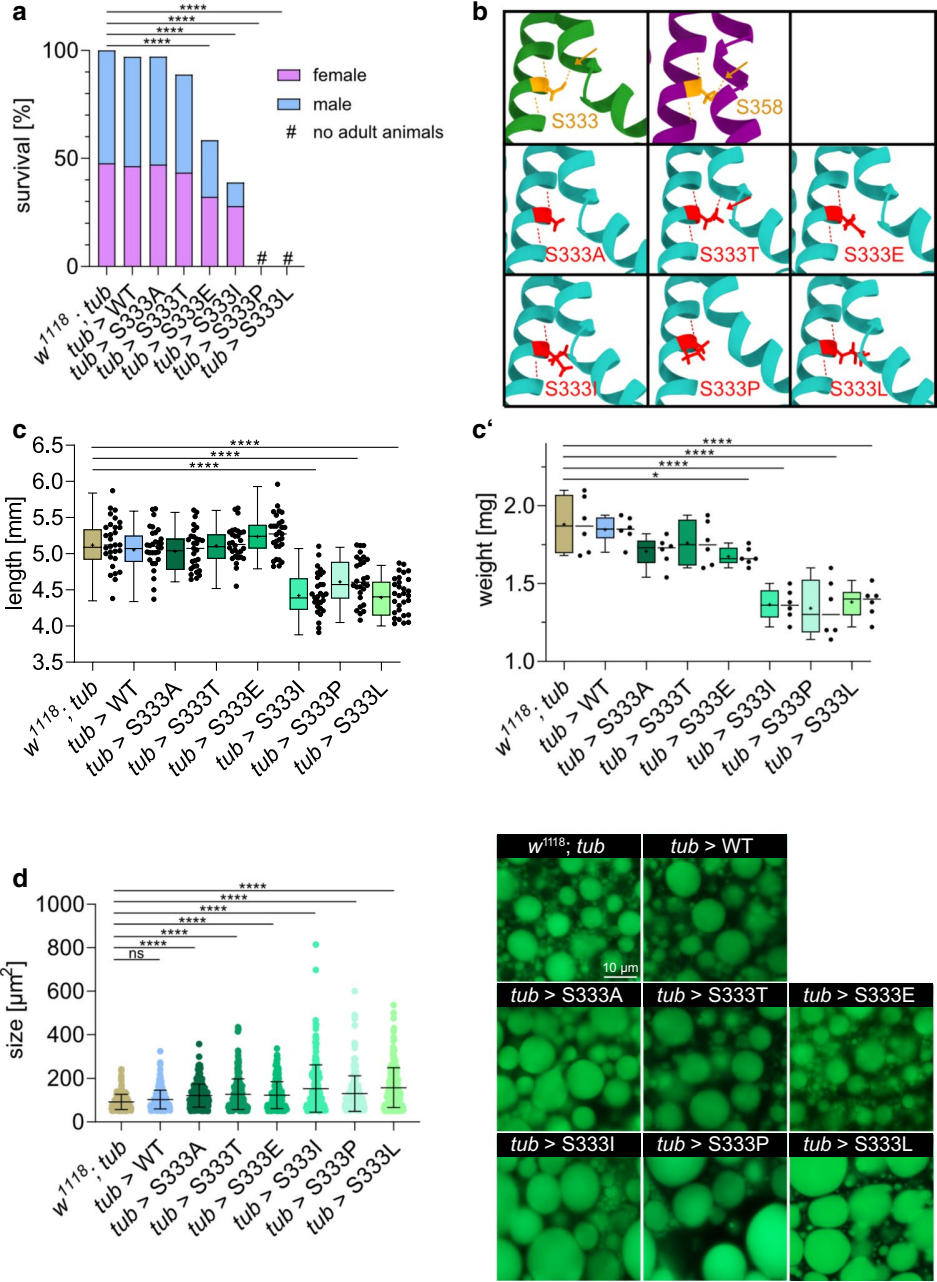
Serine-333 is crucial for the physiological functionality of CG8111. **a** Hatching assay of serine-333 variants revealed that alanine-333 (*n* = 204) and threonine-333 (*n* = 222) have no impact on survival, whereas glutamic acid-333 (*n* = 137) and isoleucine-333 (*n* = 254) show decreased survival, especially for male animals, compared with the controls (*n* = 226 and *n* = 270). Proline-333 (*n* = 218) causes a similar phenotype as leucine-333 (*n* = 222). Animals died during development and no adult animals hatch. **b** AlphaFold prediction of serine-333 variants and hydrogen bond formation are shown (arrow = hydrogen bond formed by serine-333/358 and threonine-333). For further details, see Supp. Figure 5. **c, c’** Animals that express the variant glutamic acid-333 show reduced body weight but are normal in size. Isoleucine-333, proline-333, and leucine-333 cause decreased body size and weight of third larvae. **d** All serine-333 variants show an increased size of lipid droplets in fat body tissue compared with the control. Data are shown as bars (**a**), box and whiskers (**c**, **c’**), or as scatter dot plot with mean + SD (**d**). The median is indicated by the centre line of the boxplot; upper and lower bounds indicate 75th and 25th percentiles, respectively; whiskers indicate the minimum and maximum. The average is shown by + . Statistical tests: one-way ANOVA, followed by Dunnett’s multiple comparison test (**a**, **c**, **c’**), and Kruskal–Wallis test, followed by Dunn’s multiple comparison test (**d**) were performed. *p ≤ 0.05, ***p ≤ 0.001, ****p < 0.0001

Serine-333 in CG8111 is located within the highly conserved third TM helix of the protein. To further elucidate the structural role of serine-333, we generated and analysed a series of model variants in *Drosophila* CG8111. We replaced serine-333 for threonine (hydroxyl group in the side chain, phosphorylatable), alanine (non-polar, lacking the hydroxyl group), proline (non-polar, impairs helix formation), leucine (homologue of TMEM43 p.S358L), isoleucine (non-polar) or glutamic acid (acidic, phosphomimetic). Transgenic *Drosophila* were established for all these variants to generate *UAS*-CG8111^p.S333T^, *UAS*-CG8111^p.S333A^, *UAS*-CG8111^p.S333P^, *UAS*-CG8111^p.S333I^ and *UAS*-CG8111^p.S333E^, respectively. The mutant constructs were ubiquitously expressed using *tub*-GAL4 as a driver. A Western blot analysis confirmed that all transgenic constructs were stably produced at similar levels (Supplemental Fig. 1c). Subsequently, we analysed the animals for growth, body mass and viability. We found that the substitution of serine-333 for alanine or threonine had no detrimental effects on the animal. However, isoleucine, glutamic acid, and proline at position p.333 in CG8111 severely affected the survival rate of corresponding animals. Proline impaired survival to a similar extent as leucine. Animals carrying proline-333 survived until the pupal stage but failed to hatch. Isoleucine-333 had a milder phenotype than leucine-333: About 70% of third larvae were able to pupate, but only 30% developed into adults and hatched. Interestingly, only one-third of the hatched adults were males, again indicating a stronger effect in male specimen than in females. The substitution of serine-333 for glutamic acid led to decreased survival in both females and males. Around 60% of animals hatched, with an equal distribution of male and female flies (Fig. 7a). In addition to a reduced survival, we observed that animals were smaller and lighter, when they carried isoleucine-333 or proline-333, but not for alanine-333 or threonine-333. Interestingly, glutamic acid-333 did not affect the size of the animals, however, weight was significantly reduced in a respective specimen (Fig. 7c, c’). Further analyses revealed an increased size of lipid droplets in fat bodies of all serine-333 mutant variants compared with control animals (Fig. 7d).

To understand the distinct effect of the individual mutant variants in more detail, we performed AlphaFold-based structural predictions of all serine-333 variants. The resulting datasets revealed slight conformational changes of TM4 in isoleucine-333, proline-333, leucine-333, and glutamic acid-333 variants, which may correlate with the observed lethality (Supplemental Fig. 5). Of note, the hydrogen bond, formed by serine-333, in the wild-type protein that apparently connects TM3 and TM4 remained in the threonine-333 but not in the alanine-333 variant. Thus, the presence or absence of this hydrogen bond is presumably not relevant to the observed lethality phenotypes (Fig. 7b).

## Discussion

*TMEM43* p.S358L is known to cause ARVC5. Patients carrying the mutation develop heart failure and SCD [5, 8, 12]. At present, there is no individual therapy available to prevent cardiomyopathy. However, male carriers benefit significantly from the preventive implantation of an ICD [12]. To address the disease caused by the p.S358L mutation, a deeper understanding of the underlying pathomechanisms is required. Despite several mice models investigating the mutation, such understanding is still lacking.

During recent decades, *Drosophila melanogaster* has become a standard model for the investigation of genetic and biochemical aetiologies of clinically relevant diseases [24]. TMEM43 is evolutionarily highly conserved, and CG8111 is the homologous protein present in the fruit fly. The first characterisation of CG8111 confirmed a similar membrane topology of the *Drosophila* and the human proteins (Fig. 1d) [16]. Furthermore, likewise TMEM43, CG8111 localises within the ER-membrane and the nuclear envelope with ubiquitous expression among various tissues (Fig. 2). We did not observe CG8111 at the plasma membrane, which reveals that the protein is not located at the cell surface. Our localisation experiments for CG8111 support the results of earlier studies on human TMEM43 that located the protein at the nuclear envelope and the ER [16], but do not confirm the previously stated findings with an anti- TMEM43 antibody [25]. Thus, we conclude that CG8111 is a TM-protein located at the nuclear envelope and the ER.

To assess the biological functions of CG8111, we generated and analysed ko fly lines using CRISPR Cas9-mediated targeted mutagenesis. We found that CG8111 ko flies displayed normal development, were fully viable, and had no cardiac phenotype (Fig. 3). However, CG8111 may play a fundamental biological role considering its highly conserved sequence among phylogenetically distant species, but it appears to be dispensable for the viability of flies cultured under standard laboratory conditions. This corresponds to a *Tmem43* ko mouse model that also showed no cardiopathogenic, morphological, or physiological phenotypes, even after pressure overload induced by transverse aortic constriction [13].

Further studies are needed to ascertain the function of CG8111 under stress conditions and genetic preconditions. Thus, the lack of a pathological phenotype in *CG8111* ko flies and the *Tmem43* ko mice [13] proves that the gene is dispensable for the organisms. This negligibility of CG8111 and TMEM43 to animal survival is reflected by the high allele frequency of heterozygous *TMEM43* nonsense variants in the human genome aggregation database GnomAD (https://gnomad.broadinstitute.org).

In view of these results, we tried to understand the relevant pathomechanisms by generating transgenic flies carrying the homologous *TMEM43* p.S358L mutation, which is *CG8111* p.S333L. The ubiquitous overexpression of CG8111 p.S333L caused lethality during the larval and pupal stages, while the overexpression of wild-type CG8111 had no such effects (Fig. 4a). Similar findings were previously published [11]. Here, the cardiac-specific overexpression of human TMEM43 p.S358L in transgenic mice led to severe cardiac dysfunction, fibro-fatty replacement of the myocardium, cardiomyocyte death, and decreased survival, similar to that observed in human ARVC5. However, such phenotypes were not observed in *Tmem43* p.S358L knock-in mice [13]. Based on structural predictions, the authors postulated differences in folding between the human and murine proteins [11]. Of note, our structural analysis using AlphaFold suggested a similar folding of the human TMEM43 and *Drosophila* CG8111, likely rendering *Drosophila* a suitable model to assess basic protein functionality (Fig. 1c). Furthermore, we observed clear phenotypes in response to the ubiquitous overexpression of the endogenous *Drosophila* protein CG8111 p.S333L, thus excluding some limitations present in the transgenic mice models (Fig. 4).

The variant *CG8111* p.S333L causes lethality in flies, as *TMEM43* p.S358L does in humans. However, the cause of death in both species likely differs because flies will not immediately die from sudden cardiac arrest or heart failure under normal laboratory breeding conditions. Albeit, heart failure can cause a significant reduction in *Drosophila*´s life span [26–28]. In addition to the lethality phenotype, we observed that third larvae and pupae overexpressing CG8111 p.S333L were significantly smaller and showed reduced body weight compared with animals overexpressing the wild-type protein (Fig. 4b, c). Feeding assays also revealed a diminished food intake or food utilisation (Fig. 4d). Moreover, increased size of lipid droplets in larval fat body tissue was evident, which is the main tissue for lipid storage in *Drosophila* larvae (Fig. 4e and Supplemental Fig. 3). These findings represent initial evidence for an impairment of lipid metabolism and altered lipid storage in the mutant fly. However, the TMEM43 mutation causes problems in the utilization and mobilization of lipids in adipocytes. This presumably leads to an accumulation of lipids, which may induce an “artificial” satiety signal, eventually causing the reduced food intake. Corresponding peptides mediating fat body-IPC (insulin-producing cell) communication, and thus feeding behavior, have been identified and characterized in *Drosophila* [29, 30]. Furthermore, it has been shown that reduced lipogenesis leads to increased lipid droplets in *Drosophila* and in mammalian cell culture [31, 32]. Thus, we conclude defects in lipid mobilisation for e.g. energy use. It is known that homeostasis and metabolism of cardiac lipids are crucial to proper cardiac function and lipid droplets considerably contribute to its regulation. Interestingly, it has been shown that a malfunction of cardiac lipid utilisation can cause cardiomyopathy in humans [33]. Gu et al. [15] recently showed similar findings in a murine model, where the expression of the Tmem43 mutant was associated with increased HDL and LDL, indicating a critical function of Tmem43 in lipid metabolism. Taken together, we consider an impaired lipid metabolism causative to the phenotypes we observed in response to overexpression of CG8111 p.S333L, i.e., heart arrhythmias, reduced body weight and size, a delayed larval development, and death of corresponding animals (Figs. 4, 6).

This assumption is supported by our proteomics data revealing that overexpression of CG8111 p.S333L compared with CG8111 WT resulted in an upregulation of proteins involved in fatty acid and lipid metabolism (Fig. 5 and Supplemental Fig. 4). It has been shown previously that metabolomic analysis offers a powerful instrument for investigating *Drosophila* physiology [34]. Our NMR-based metabolite analysis also showed an accumulation of fatty acids and BCAA in flies that overexpressed CG8111 p.S333L (Fig. 5 and Supplemental Fig. 4). In this regard, the fact that BCAA can inhibit fatty acid transport and utilisation in mice further supports our model [35, 36]. Together with the increased lipid droplet size in corresponding animals, as well as the concomitantly increased levels of fatty acids, it appears conceivable that high levels of BCAA are responsible for these phenotypes in CG8111 p.S333L overexpressing animals. The impaired lipid metabolism could also be causative of the fibrofatty replacement found in ARVC5 patients. We assume that the systemic metabolomic defects become prominently visible in the heart of ARVC5 patients. However, understanding the detailed correlation requires further analysis.

The ubiquitous overexpression of CG8111 p.S333L turned out to be lethal to different extents, depending on the specific type of driver. Furthermore, we found that males were more strongly affected by ubiquitous overexpression of the mutant variant than females. We suspect that different spatial and temporal expression patterns of the used ubiquitous drivers are responsible for different sex-specific survival outcomes. However, the overexpression of CG8111 p.S333L with different tissue-specific drivers did not lead to the identification of a single tissue as responsible for the lethality. A sex-specificity was also evident in animals overexpressing CG8111 p.S333L predominantly in the heart. Again, significant effects, i.e. cardiac arrhythmias, occurred only in five-week-old males, while age-matched females did not exhibit any phenotype (Fig. 6). Our results are striking considering the apparent sex-specificity of the TMEM43 p.S358L mutation. However, a mechanistic understanding of this phenomenon is currently lacking for both, the human as well as the *Drosophila* protein.

Our data demonstrate that CG8111 plays pivotal roles in the heart, as well as in other tissues, probably by ensuring proper lipid metabolism and utilisation. These fundamental cellular functions appear to be impaired by the overexpression of CG8111 p.S333L. Although our study does not conclusively explain the pathomechanism of p.S333L, we found that the impact of the mutation strongly depends on the amino acid substitute at position 333. Leucine, isoleucine, and proline led to premature death, reduced body size and weight, and increased the size of lipid droplets in the larval fat body. These strong effects may suggest that the integrity of the TM-helix was compromised by amino acids that cause steric hindrance. It has been shown that proline is a helix breaker and that isoleucine is a weak alpha-helix former [37]. Furthermore, predicted molecular structures of CG8111 with the corresponding amino acid exchanges included, support for a potentially reduced helix interaction between TM3 and TM4.

In summary, the overexpression of CG8111 p.S333L in *Drosophila* induces a remarkable physiological phenotype, which is reminiscent of the fibrofatty myocardial remodelling found in TMEM43 p.S358L hearts. While the underlying molecular pathomechanism is still not fully understood, this study introduces *Drosophila melanogaster* as a new and adequate model to further address this issue in the future. Based on our current findings, we conclude that CG8111 represents a critical determinant of lipid metabolism and utilisation, a biological function that may be conserved throughout evolution, with impairments being the physiological basis of abnormal cardiac remodelling and functionality.

## Materials and methods

### Fly stocks

The following fly stocks were used in this study: For CRISPR ko generation, transgenic gRNA (BL77060), *vas*-Cas9 (BL51323), and balancer stock *w*^1118^; TM3/TM6b were used. The driver lines were *act5c*-GAL4 [38], *da*-GAL4 (BL55849), *tub*-GAL4 (BL5138), *48Y*-GAL4 (BL4935), *cg25c*-GAL4 (BL7011), *elav*-GAL4 (BL458), *handC*-GAL4 8.0 [39], *mef2*-GAL4 (BL27390), *repo*-GAL4 (BL7415), and *sgs3*-GAL4 (BL6870). To generate UAS- and GAL4-control animals, crosses with the respective lines and *w*^1118^ (BL5905) were performed.

### Generation of CRISPR/Cas9-induced *CG8111* ko flies

Two approaches were conducted to generate *CG8111* ko animals: injection of gRNAs into a Cas9 strain and crossing transgenic fly lines carrying either the gRNA or Cas9 construct. For both approaches, flies were tested by PCR for positive CRISPR events. Siblings with no CRISPR event were used as controls. Target sites for gRNA design were selected and evaluated with the online tool “Optimal Target Finder” [40].

Transgenic target site: GCGAAACACTTCGTAGCCACTGG.

Target sites for injection: CACGGAGCTCTACTGGAACGAGG, GGTGAACGATTCTGCCCTCGAGG, CCAAATGTACCAGTGGGTCGAGG, CTTTCGATGGGAAAGTGACTGGG, GGCACCCGATCCTCAGTTCCCGG, GATCGGCGCCTGCCTCTTACTGG.

Respective DNA templates for T7-mediated in vitro transcription of gRNAs were synthesized at Biolegio (Nijmegen, Netherlands). The backbone was designed as previously described [41]:

TAATACGACTCACTATAGtarget_site_sequenceGTTTTAGAGCTAGAAATAGCAAGTTAAAATAAGGCTAGTCCGTTATCAACTTGAAAAAGTGGCACCGAGTCGGTGCTTTT. To generate double-stranded DNA templates, 100 µM of complementary oligonucleotides were mixed and heated at 95 °C for 5 min. Afterwards, the templates were allowed to cool down at room temperature. Commercial in vitro transcription and injection was performed by The BestGene Inc. (Chino Hills, USA). The positions of target sites are shown in Fig. 3a.

### Transgenic lines

*UAS*-CG8111, *UAS*-CG8111::HA, mutant forms, and model variants of the gene were assembled in *Escherichia coli*/*Saccharomyces cerevisiae*/*Drosophila melanogaster* triple-shuttle derivatives (YED-vectors) of the pUAST vector [42] that were adapted for cloning by homologous recombination in vivo. The YED-vectors with the different variants were constructed using appropriate PCR products or synthesized DNA fragments as described, previously [43]. Fly husbandry was carried out as described previously [44]. For the site-specific integration into the genome, modified versions of the YED-vectors were used to ensure identical insertion loci for comparable expression levels. The resulting constructs were injected at BestGene (Chino Hills, CA, USA) to generate the transgenic lines.

### Developmental assay

To synchronise development, flies were allowed to lay embryos for 2 h on fruit juice agar plates with yeast paste. Afterwards, flies were transferred to another fruit juice agar plate with yeast paste and were allowed to lay embryos for another 2 h. Only embryos of the second plate were counted and transferred to a new fruit juice agar plate. After 24 h, the first hatched larvae were counted and transferred to small vials containing fly food. Several days later, the pupated larvae at the wall of the vial were counted. Finally, the hatched flies were counted.

### Lifespan assay

The flies were collected (Mondays, Wednesdays, and Fridays) one to three days after hatching, and the males and females were separated. During the experiment, the flies were kept in cages in a climate chamber at 25 °C, 65% humidity, and on a 12-h dark/12-h light cycle. Animals received fresh food every 2–3 days. Dead flies were counted each time fresh food was offered. Flies that were sticking to the food but were not dead were excluded from the experiment.

### Hatching assay

Flies were allowed to lay embryos for 24 h on fruit juice agar plates with yeast paste. Another 24 h later, the first larvae were counted and transferred to medium-sized vials containing fly food. As soon as the flies had hatched, the number of females and males were counted until all pupae were empty or dead.

### Feeding assay

Flies were allowed to lay embryos for 8 h. After another 72 h, third instar larvae were washed out of the fly food and starved for one hour in 20% sucrose in 1 × PBS. Subsequently, animals were transferred to agar plates supplemented with 2.5% brilliant blue yeast paste (or yeast paste as a control). Larvae were allowed to feed for 30 min. Then, the animals were washed with 1% Triton in 1 × PBS to remove the remaining yeast on the outside of the larvae. Animals were collected in cohorts of five and were homogenised in 1% Triton in 1 × PBS. Samples were centrifuged for one min at 13,000*g* to remove insoluble parts from the solution. Finally, absorption at 610 nm was measured with a NanoDrop spectrophotometer (control animals were used for blanking).

### Measuring length and weight of third larvae and pupae

To measure the weight, larvae or pupae were collected in cohorts of five animals. Their weight was measured with a microscale, and the average weight of one animal was calculated. To immobilise and stretch the larvae, the animals were incubated in 60 °C hot water for 10 s. Afterwards, animals were placed on a microscope slide and pictures were taken. Subsequently, their length was calculated using Affinity Photo.

### Semi-automated optical heartbeat analysis

*handC*-GAL4 was used to induce the expression of CG8111^p.S333L^, CG8111^WT^, and various controls in heart cells. Characteristic heart parameters, including heart rate, heart period, systolic and diastolic interval, fractional shortening, and rhythmicity were measured via semi-automatic optical heartbeat analysis, SOHA [45, 46]. For the experiments, 1- or 5-week-old male and female flies were used.

### Generation of antibodies

A 402 bp sequence, corresponding to the loop encoding region (aa 34-168), was amplified by PCR and cloned into the pET-29b vector (Novagen, Madison, WI) to generate a His-tag fusion construct. Protein expression was induced with 1 mM IPTG in *E. coli* Rosetta™ (DE3). After protein isolation from 5 l overnight cultures, and subsequent Ni–NTA-affinity chromatography, protein purity was analysed by SDS-PAGE and the corresponding eluates were used for antibody production by a commercial service (Pineda antibody service, Berlin, Germany). The resulting antisera were affinity-purified, and the specificity of the antibodies was confirmed via immunoblotting using protein extracts from wild-type controls and CG8111 heterozygous and homozygous mutants.

### Immunohistochemistry

Immunostainings with *Sf*21 cells and third instar larvae were performed as described previously [47, 48]. The antibodies used in this study were as follows: primary antibodies: rabbit anti-HA (1:100, Sigma) and mouse anti-Calnexin (1:200, DSHB, 99A-6-2-1); secondary antibodies: goat anti-mouse-Cy3 (1:200, Dianova), goat anti-rabbit-AF633 (1:100, Invitrogen). Confocal images were captured with a laser scanning microscope (Zeiss LSM800). Image processing was done with Fiji and Affinity Photo (Serif).

### Topology analysis of CG8111 in S2 cells

To determine the orientation of CG8111 in the ER-membrane, redox-sensitive GFP (roGFP) was used [20]. Cultured *Drosophila* S2 cells were transfected with either C-terminal or N-terminal roGFP-tagged versions of CG8111^WT^. Another construct in which the loop region between TM1 and TM2 of CG8111 had been exchanged with roGFP was established. In addition, a C-terminal roGFP tagged version of CG8111^p.S333L^ was generated. All constructs were cloned into the pAc5.1 vector. We used TransFectin™ (BioRad, Hercules, CA, USA), for the transfection of S2 cells. Cells were grown on round cover slips (12 mm diameter) in 6-well plates for 72 h at 28 °C. The coverslip was placed in a homemade imaging chamber, which was filled with 1 × PBS. Images were taken with a Zeiss LSM800 and Zen Blue 2.6 software (Zeiss, Jena, Germany). Samples were excited with either a 405 nm laser or a 488 nm laser. GFP emissions were recorded with the 20× objective lens (Zeiss, EC-Plan-Neofluar 20×/0.5). Afterwards, the PBS was replaced by Diamide (0.5 mM) and incubated for at least 1 min for complete sample oxidation. Subsequently, pictures were taken with the same microscopic settings as used for the first set of images. Then, the Diamide solution was exchanged with DTT (2 mM) to induce reducing conditions. The DTT solution was incubated for at least 7 min, and pictures were again captured. Data evaluation was done using ImageJ (Fiji) to measure the mean pixel intensity of individual transfected S2 cells under the described conditions to calculate an intensity ratio (405 nm/488 nm). The intensity ratio of the steady state was compared with that of the oxidised and reduced state, respectively, to deduce the orientation and topology of the CG8111 fusion protein.

### Lipid droplet analysis

Third larvae were prepared to access the fat body. Tissue was fixed for 30 min in 4% formaldehyde with agitation and afterwards washed with 1 × PBS several times. LDs were stained with BODIPY 493/503 (Invitrogen) for 30 min, with slight agitation and in darkness. After washing for 10 min, the tissue was embedded in Fluoromount/DAPI and analysed under a Zeiss LSM800. Images were captured with a Zeiss Apo 40 × lens and analysed for LD number and size using Fiji image analysis software [49]. For statistical analyses, 10 animals were stained and analysed per genotype.

### Western blot

SDS-PAGE and Western blotting were performed using standard protocols. The primary antibodies used were rabbit anti-CG8111 (1:100), and mouse anti-HA (1:1000, DSHB). The secondary antibodies were IRDye® 680 RD goat anti-rabbit (1:5,000, LiCor), and IRDye® 800 CW goat anti-mouse (1:5,000, LiCor). The membranes were imaged with an Odyssey Clx Imager (LiCor, Lincoln; NE, U.S.A.), and quantification was performed using Empria Software (LiCor).

### NMR metabolomics

Third instar larvae were grouped into genotype-specific cohorts of 30 animals, and five cohorts per genotype were independently analysed to assess metabolite composition. Briefly, the animals (50 mg/cohort) were homogenized in 250 µl ice-cold methanol, 70 µl H_2_O, and 125 µl chloroform. Afterwards, samples were vigorously shaken, and an additional 125 µl chloroform and 100 µl H_2_O were added. After incubation on ice (15 min), the samples were centrifuged (10,000 × *g*, 15 min, 4 °C). The resulting aqueous phase was lyophilized, and the chloroform of the organic phase was evaporated under vacuum. Samples were stored at − 80 °C. To remove the signal from residual methanol from the aqueous phase, 50 µl of deuterated methanol was added to each sample before the samples were lyophilised again. Immediately before NMR measurements, the aqueous phase was rehydrated in 200 µl of 37.5 mM phosphate buffer (pD 6.95) in D_2_O by shaking at 800 rpm at 22 °C for 45 min. The buffer contained 0.747 mM of the chemical shift reference (Trimethylsilyl)propionic-2,2,3,3-d4 acid, sodium salt (TSPd4), and 0.05% w/v of sodium azide to prevent bacterial growth. Similarly, the organic phase was dissolved in 200 µl of deuterated chloroform containing 0.1% tetramethyl silane (TMS). For each sample, 180 μl was transferred to a 3 mm NMR tube The NMR measurements were carried out at 25 °C on a Bruker Avance-III 700 spectrometer (Bruker Biospin, Germany) operating at a ^1^H frequency of 700.20 MHz and equipped with a and 5 mm QCI cryoprobe. The ^1^H NMR spectra were acquired using a single 90° pulse experiment. For the aqueous samples, the water signal was suppressed by excitation sculpting with perfect echo [50], and a total of 64 k data points spanning a spectral width of 20 ppm were collected in 128 transients. For the organic samples, a total of 32 k data points spanning a spectral width of 15.9 ppm were collected in 128 transients. For assignment purposes, two-dimensional ^1^H-^1^H TOCSY and ^1^H-^13^C HSQC spectra were acquired. The spectra were processed using iNMR (www.inmr.net). An exponential line broadening of 0.5 Hz was applied to the free induction decay prior to Fourier transformation. All spectra were referenced to the DSS signal at 0 ppm, automatically phased, and baseline corrected. The spectra were aligned using icoshift [51], and the region around the residual water signal (4.88–4.67 ppm) was removed. The spectra were normalized by probabilistic quotient area normalization [52], and the data were scaled using Pareto scaling [53] and centred.

### NMR data analysis

Initially, the whole dataset was subjected to principal component analysis (PCA) [54]. Afterwards, orthogonal projection to latent structures discriminant analysis (OPLS-DA) models were created to separate the larvae expressing CG8111^p.S333L^ from those expressing CG8111^wt^ and the control larvae. OPLS-DA models are multivariate models that predict group membership based on multivariate input, in this case, the NMR spectra. The model separates variations due to group membership from other (orthogonal) variations [55]. The OPLS-DA models were validated by cross validation, where models were made with randomly chosen groups of samples left out one at a time, and group membership was predicted for the left-out samples. The predictability (*Q*^2^) of the models was 0.71 for the aqueous samples and 0.86 for the organic samples, respectively, indicating high-quality models. Significant spectral correlations were identified by applying sequential Bonferroni correction (*P* < 0.05) for an assumed total number of 100 metabolites. The correlations were performed in MATLAB (The MathWorks, Natick, MA). Signal assignments were based on chemical shifts using earlier assignments and spectral databases described elsewhere [56–58]. All multivariate analyses were performed using the Simca-P software (Umetrics, Sweden).

### Proteome analysis

Third instar larvae were grouped into genotype-specific cohorts of five animals, and four cohorts per genotype were independently analysed to assess the proteome composition. First, the animals were frozen in liquid nitrogen and ground to powder. The samples were further processed according to the manufacturer’s protocol for the iST Preomics Kit (PREOMICS GMBH, Planegg/Martinsried, Germany).

#### Mass spectrometry

Reversed-phase chromatography was performed using the UltiMate 3000 RSLCnano System (Thermo Fisher Scientific). The samples were loaded onto a trap column (Acclaim PepMap 100 C18, 5 µm, 0.1 mm × 20 mm, Thermo Fisher Scientific) and washed with loading buffer (0.1% TFA in H_2_O) at a flow rate of 25 µl/min. The trap column was switched in line with a separation column (Acclaim PepMap 100 C18 2 µm, 0.075 mm × 150 mm, Thermo Fisher Scientific). Subsequently, bound peptides were eluted by changing the mixture of buffer A (99% water, 1% acetonitrile, 0.1% formic acid) and buffer B (80% acetonitrile, 20% water and 0.1% formic acid) from 100:0 to 20:80 within 180 min. The flow rate was kept constant at 0.3 µl/min. Eluted compounds were directly electrosprayed through an EASY-Spray ion source (Thermo Fisher Scientific) into a Q Exactive Plus Orbitrap mass spectrometer (Thermo Fisher Scientific). Eluates were analysed by measuring the masses of the intact molecules as well as the masses of the fragments, which were generated by higher-energy collisional dissociation (HCD) of the corresponding parent ion. For the analysis, PEAKS Online (Version 1.6, Bioinformatics Solutions Inc., Waterloo, Canada), in combination with a *Drosophila*-specific TrEMBL database, was used to determine peptide-specific amino acid sequences (parent mass error tolerance: 15 ppm; fragment mass error tolerance: 0.25 Da; enzyme: trypsin; max missed cleavages: 2; selected PTMs: carbamidomethylation, oxidation). Label-free quantification was performed by comparing peptide and protein amounts of different groups according to established protocols [59], with each group consisting of four independent biological replicates. The protein list was controlled by FDR (threshold: 1%) and significance was calculated by PEAKS online (one-way ANOVA). Classification as a possible interaction partner required *P* < 0.05, with quantification being based on at least three individual protein-specific peptides.

### Statistical analysis

For the statistical analysis, Graph Pad Prism 9 was used. Datasets were analysed for normal distribution using the D’Agostino & Pearson test. If normally distributed, the one-way ANOVA test was used, otherwise the Kruskal–Wallis test was applied. If only two datasets were compared, an unpaired t-test was done. Life span data were analysed by log-rank (Mantel–Cox) tests.

## Limitations

In this study, we investigated an organism which is phylogenetically distant from mammals. However, because TMEM43 is highly conserved, our study provides information about the cellular and molecular functions of this ancient protein. *Drosophila* can survive without a functional heart; therefore, SCD is not detectable in flies. This is one of the biggest advantages of the *Drosophila* model. Even in heart failure mutants, and in mutants with severe morphological malformation of the heart, the organ is still accessible to physiological and genetic treatments. In sum, we observed in adult CG8111 p.S333L mutant flies an arrhythmic phenotype that may mimic clinical arrhythmias found in ARVC5 patients.

### Supplementary Information

Below is the link to the electronic supplementary material.

Supplementary file1 (MP4 2879 kb)

Supplementary file2 (DOCX 4687 kb)

## Data Availability

The datasets generated during and/or analysed during the current study are available from the corresponding author on request.

## Abbreviation

DSHB: Developmental Studies Hybridoma Bank, Iowa, US

## References

[1] Paul M (2009). Genes causing inherited forms of cardiomyopathies: a current compendium. Herz.

[2] Muthappan P, Calkins H (2008). Arrhythmogenic right ventricular dysplasia. Prog Cardiovasc Dis.

[3] Pilichou K (2011). Arrhythmogenic cardiomyopathy: transgenic animal models provide novel insights into disease pathobiology. Circ Cardiovasc Genet.

[4] Dreger M (2001). Nuclear envelope proteomics: novel integral membrane proteins of the inner nuclear membrane. Proc Natl Acad Sci (USA).

[5] Merner ND (2008). Arrhythmogenic Right ventricular cardiomyopathy type 5 Is a fully penetrant, lethal arrhythmic disorder caused by a missense mutation in the TMEM43 gene. Am J Hum Genet.

[6] Milting H (2015). The TMEM43 Newfoundland mutation p.S358L causing ARVC-5 was imported from Europe and increases the stiffness of the cell nucleus. Eur Heart J.

[7] Dominguez F (2020). Clinical characteristics and determinants of the phenotype in TMEM43 arrhythmogenic right ventricular cardiomyopathy type 5. Heart Rhythm.

[8] Christensen AH (2011). Mutation analysis and evaluation of the cardiac localization of TMEM43 in arrhythmogenic right ventricular cardiomyopathy. Clin Genet.

[9] Baskin B (2013). TMEM43 mutations associated with arrhythmogenic right ventricular cardiomyopathy in non-Newfoundland populations. Hum Genet.

[10] Haywood AFM (2013). Recurrent missense mutations in TMEM43 (ARVD5) due to founder effects cause arrhythmogenic cardiomyopathies in the UK and Canada. Eur Heart J.

[11] Padrón-Barthe L (2019). Severe cardiac dysfunction and death caused by arrhythmogenic right ventricular cardiomyopathy type 5 are improved by inhibition of glycogen synthase kinase-3β. Circulation.

[12] Hodgkinson KA (2016). Long-term clinical outcome of arrhythmogenic right ventricular cardiomyopathy in individuals with a pS358L mutation in TMEM43 following implantable cardioverter defibrillator therapy. Circul Arrhythm Electrophysiol.

[13] Stroud MJ (2017). Luma is not essential for murine cardiac development and function. Cardiovasc Res.

[14] Zheng G (2018). TMEM43-S358L mutation enhances NF-κB-TGFβ signal cascade in arrhythmogenic right ventricular dysplasia/cardiomyopathy. Protein Cell.

[15] Gu Q (2021). Systems genetics analysis defines importance of TMEM43/LUMA for cardiac and metabolic related pathways. Physiol Genomics.

[16] Bengtsson L, Otto H (2008). LUMA interacts with emerin and influences its distribution at the inner nuclear membrane. J Cell Sci.

[17] Hu Y (2011). An integrative approach to ortholog prediction for disease-focused and other functional studies. BMC Bioinformatics.

[18] Flybase TF (1999). database of the *Drosophila* genome projects and community literature. Nucleic Acids Res.

[19] Jumper J (2021). Highly accurate protein structure prediction with AlphaFold. Nature.

[20] Tsachaki M (2015). Determination of the topology of endoplasmic reticulum membrane proteins using redox-sensitive green-fluorescence protein fusions. Biochim Biophys Acta BBA Mol Cell Res.

[21] Chintapalli VR, Wang J, Dow JA (2007). Using FlyAtlas to identify better *Drosophila melanogaster* models of human disease. Nat Genet.

[22] Zirin J (2020). Large-scale transgenic *Drosophila* resource collections for loss- and gain-of-function studies. Genetics.

[23] Ge SX, Jung D, Yao R (2020). ShinyGO: a graphical gene-set enrichment tool for animals and plants. Bioinformatics.

[24] Souidi A, Jagla K (2021). *Drosophila* heart as a model for cardiac development and diseases. Cells.

[25] Franke WW (2014). Protein LUMA is a cytoplasmic plaque constituent of various epithelial adherens junctions and composite junctions of myocardial intercalated disks: a unifying finding for cell biology and cardiology. Cell Tissue Res.

[26] Melkani GC (2011). The UNC-45 chaperone is critical for establishing myosin-based myofibrillar organization and cardiac contractility in the *Drosophila* heart model. PLoS ONE.

[27] Neely GG (2010). A global *in vivo Drosophila* RNAi screen identifies NOT3 as a conserved regulator of heart function. Cell.

[28] Drechsler M, Schmidt A, Paululat A (2013). The conserved ADAMTS-like protein Lonely heart mediates matrix formation and cardiac tissue integrity. PLoS Genet.

[29] Geminard C, Rulifson EJ, Leopold P (2009). Remote control of insulin secretion by fat cells in *Drosophila*. Cell Metab.

[30] Delanoue R (2016). *Drosophila* insulin release is triggered by adipose Stunted ligand to brain Methuselah receptor. Science.

[31] Schott MB (2019). Lipid droplet size directs lipolysis and lipophagy catabolism in hepatocytes. J Cell Biol.

[32] Gronke S (2005). Brummer lipase is an evolutionary conserved fat storage regulator in *Drosophila*. Cell Metab.

[33] Schulze PC, Drosatos K, Goldberg IJ (2016). Lipid use and misuse by the heart. Circ Res.

[34] Cox JE, Thummel CS, Tennessen JM (2017). Metabolomic studies in *Drosophila*. Genetics.

[35] Nishimura J (2010). Isoleucine prevents the accumulation of tissue triglycerides and upregulates the expression of PPARalpha and uncoupling protein in diet-induced obese mice. J Nutr.

[36] Huang Y (2011). Branched-chain amino acid metabolism in heart disease: an epiphenomenon or a real culprit?. Cardiovasc Res.

[37] Chou PY, Fasman GD (1978). Prediction of the secondary structure of proteins from their amino acid sequence. Adv Enzymol Relat Areas Mol Biol.

[38] Storkebaum E (2009). Dominant mutations in the tyrosyl-tRNA synthetase gene recapitulate in *Drosophila* features of human Charcot-Marie-Tooth neuropathy. Proc Natl Acad Sci.

[39] Sellin J (2006). Dynamics of heart differentiation, visualized utilizing heart enhancer elements of the *Drosophila melanogaster* bHLH transcription factor Hand. Gene Expr Patterns.

[40] Gratz SJ (2014). Highly specific and efficient CRISPR/Cas9-catalyzed homology-directed repair in *Drosophila*. Genetics.

[41] Böttcher R (2014). Efficient chromosomal gene modification with CRISPR/cas9 and PCR-based homologous recombination donors in cultured *Drosophila* cells. Nucleic Acids Res.

[42] Brand AH, Perrimon N (1993). Targeted gene expression as a means of altering cell fates and generating dominant phenotypes. Development.

[43] Paululat A, Heinisch JJ (2012). New yeast/*E. coli*/*Drosophila* triple shuttle vectors for efficient generation of *Drosophila* P element transformation constructs. Gene.

[44] Wang S (2012). GBF1 (Gartenzwerg)-dependent secretion is required for *Drosophila* tubulogenesis. J Cell Sci.

[45] Fink M (2009). A new method for detection and quantification of heartbeat parameters in *Drosophila*, zebrafish, and embryonic mouse hearts. Biotechniques.

[46] Cammarato A, Ocorr S, Ocorr K (2015). Enhanced assessment of contractile dynamics in *Drosophila* hearts. Biotechniques.

[47] Hallier B (2016). *Drosophila* neprilysins control insulin signaling and food intake via cleavage of regulatory peptides. Elife.

[48] Meyer H (2009). Neprilysin 4, a novel endopeptidase from *Drosophila melanogaster*, displays distinct substrate specificities and exceptional solubility states. J Exp Biol.

[49] Schindelin J (2012). Fiji: an open-source platform for biological-image analysis. Nat Methods.

[50] Adams RW (2013). “Perfecting” WATERGATE: clean proton NMR spectra from aqueous solution. Chem Commun (Camb).

[51] Savorani F, Tomasi G, Engelsen SB (2010). icoshift: a versatile tool for the rapid alignment of 1D NMR spectra. J Magn Resonance (San Diego, Calif, 1997).

[52] Dieterle F (2006). Probabilistic quotient normalization as robust method to account for dilution of complex biological mixtures. Application in1H NMR metabonomics. Anal Chem.

[53] Craig A (2006). Scaling and normalization effects in NMR spectroscopic metabonomic data sets. Anal Chem.

[54] Stoyanova R, Brown TR (2001). NMR spectral quantitation by principal component analysis. NMR Biomed.

[55] Bylesjo M (2006). MASQOT-GUI: spot quality assessment for the two-channel microarray platform. Bioinf (Oxf, Engl).

[56] Rohde PD (2021). Prediction of complex phenotypes using the *Drosophila melanogaster* metabolome. Heredity (Edinb).

[57] Malmendal A (2006). Metabolomic profiling of heat stress: Hardening and recovery of homeostasis in *Drosophila*. Am J Physiol Regul Integr Compara Physiol.

[58] Pedersen KS (2008). Metabolomic signatures of inbreeding at benign and stressful temperatures in *Drosophila melanogaster*. Genetics.

[59] Lin H, He L, Ma B (2013). A combinatorial approach to the peptide feature matching problem for label-free quantification. Bioinf (Oxf, Engl).

