# Supplementary material for: A Drosophila melanogaster model for TMEM43-related arrhythmogenic right ventricular cardiomyopathy type 5
Source: Cell Mol Life Sci. 2022 Jul 22;79(8):444. doi: 10.1007/s00018-022-04458-0 (PMC9307560; doi:10.1007/s00018-022-04458-0)
Supplement: Supplementary file 2 — Supplementary file2 (DOCX 4687 kb) [file 18_2022_4458_MOESM2_ESM.docx]

Supplementary Materials for

# A *Drosophila melanogaster* model for *TMEM43* related Arrhythmogenic right ventricular cardiomyopathy type 5

**Nora Klinke, Heiko Meyer, Sandra Ratnavadivel, Marcel Reinhardt, Jürgen J. Heinisch, Anders Malmendal, Hendrik Milting, and Achim Paululat**

*Corresponding author. Email: : [achim.paululat@uos.de](mailto:achim.paululat@uos.de)

**This PDF file includes:**

Figs. S1 to S5

Table S1

**Other Supplementary Materials for this manuscript include the following:**

Movie S1


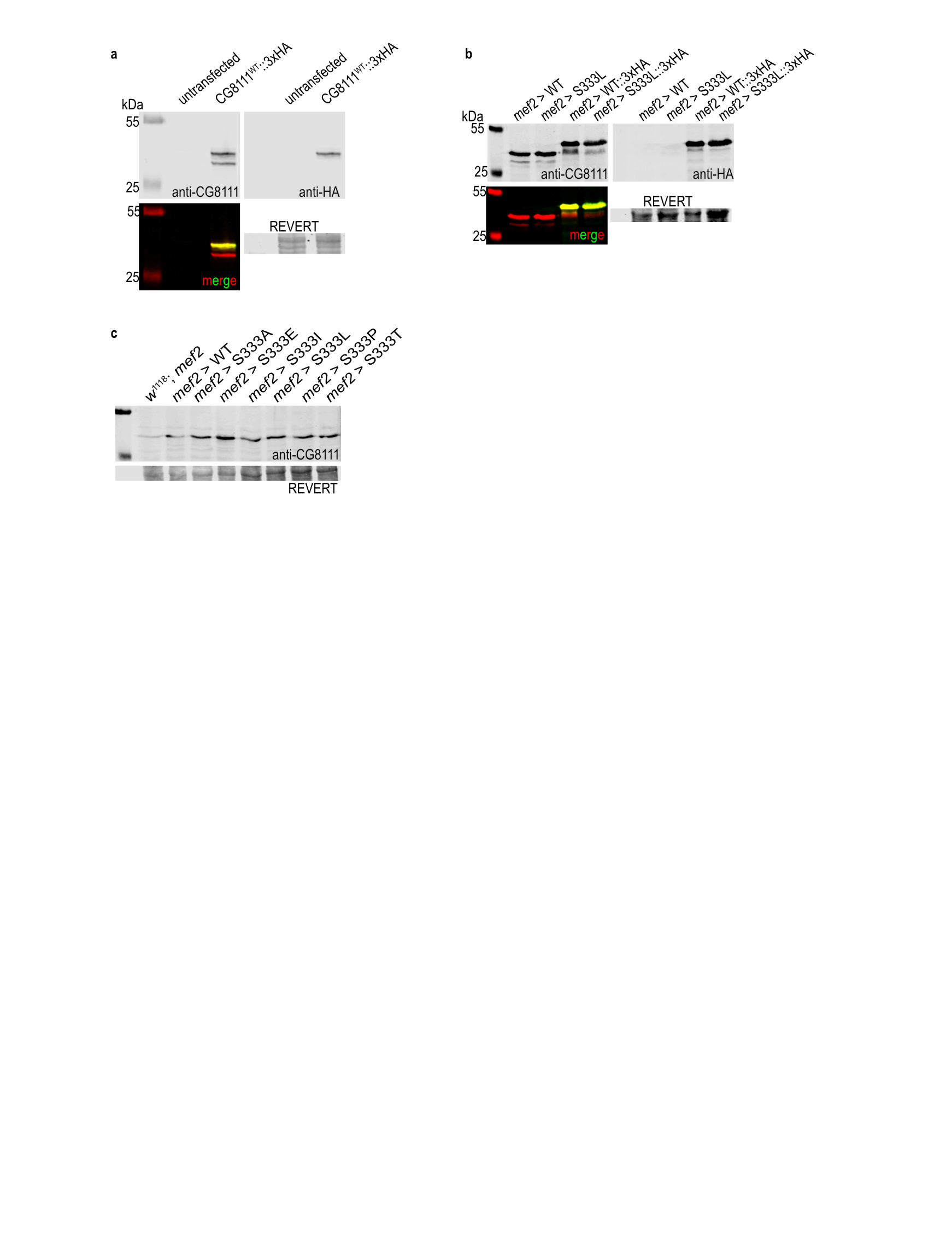


**Supplemental figure 1:** Expression validation by Western blot analysis

**a:** Western Blot of Sf21-cells infected with CG8111::HA showed specificity of the antibody raised against CG8111. **b:** *UAS*-CG8111^WT^ and *UAS*-CG8111^S333L^ constructs (tagged and untagged) were expressed at similar levels in flies. **c:** Western blot analysis of CG8111 S-333 variants revealed similar expression levels of all constructs.


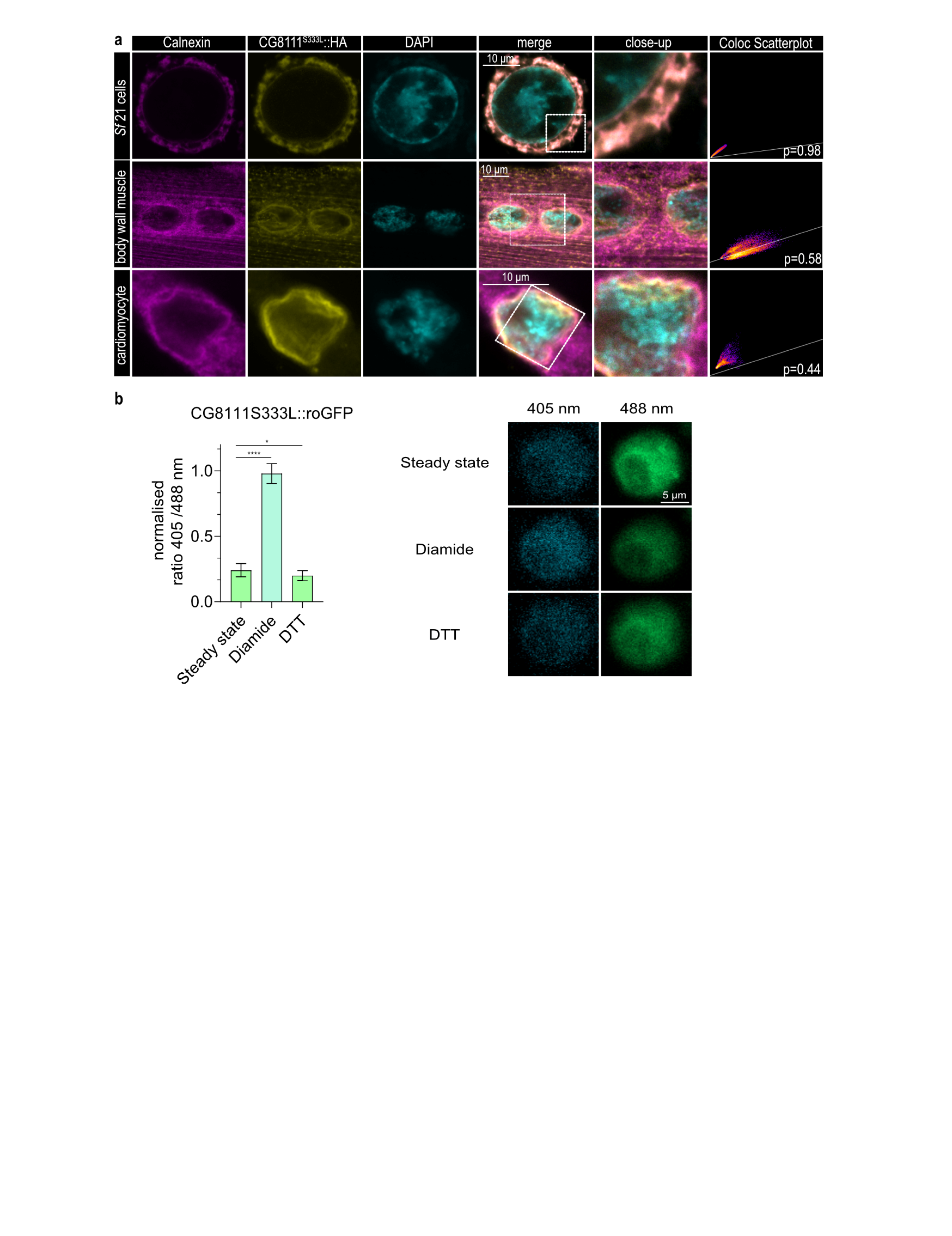


**Supplemental figure 2:** CG8111p.S333L showed similar localization and topology as the wild‑type protein

**a:** *Drosophila* CG8111^S333L^ localised to the ER compartment and the nuclear membrane in Sf21-cells, muscle tissue and cardiomyocytes. Colocalisation of CG8111 and Calnexin was calculated by Pearson’s correlation coefficient. **b:** Orientation assay of CG8111^S333L^ revealed same topology for the C-terminus in the ER-membrane compared with the wild-type protein. Data shown as mean with SD. Statistical test: Kruskal-Wallis test, followed by Dunnett’s Multiple Comparison Test. * = p≤0.05, **** = p<0.0001


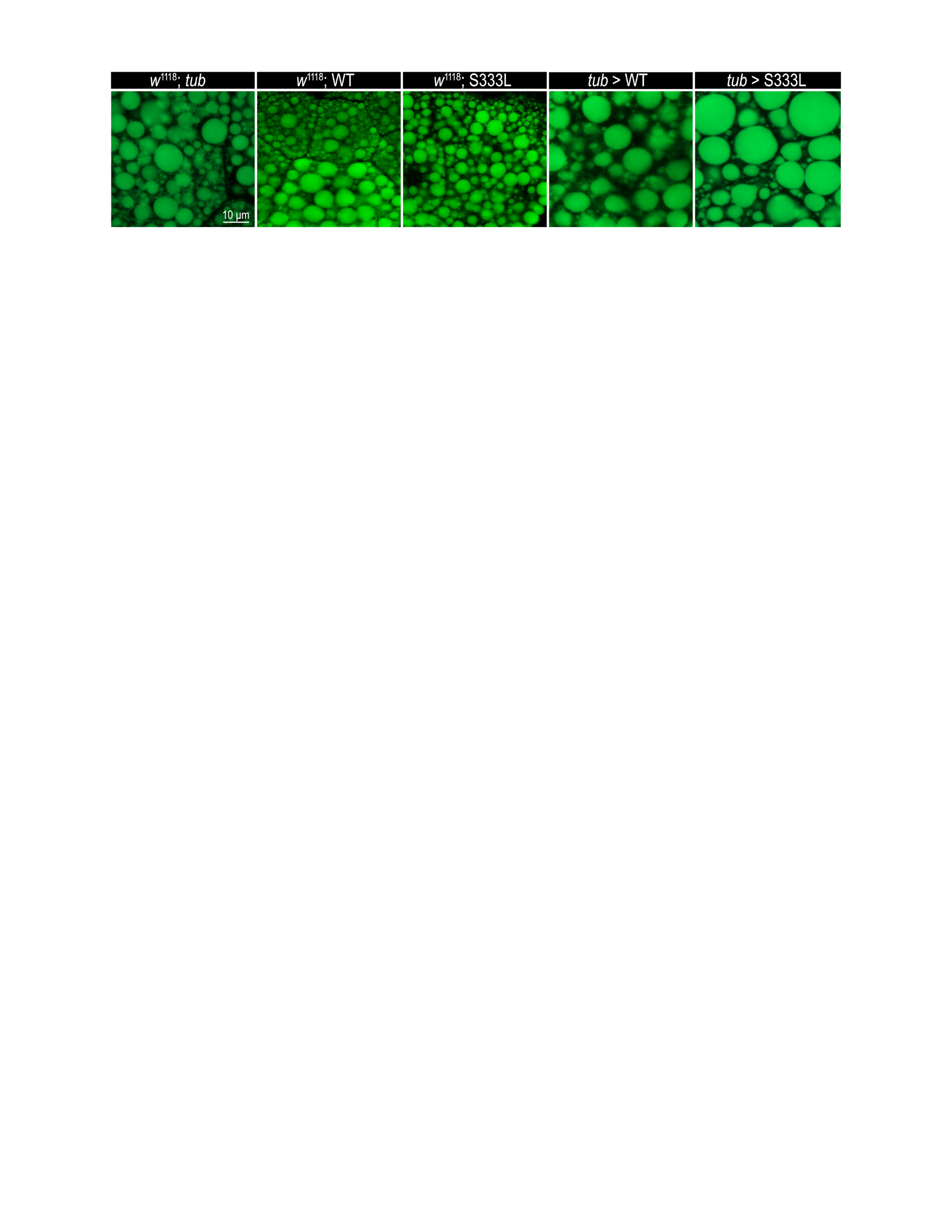


**Supplemental figure 3:** Lipid droplet size is increased in animals overexpressing CG8111^S333L^

Lipid droplet size was increased in wandering third larvae overexpressing the point mutation variant of CG8111.


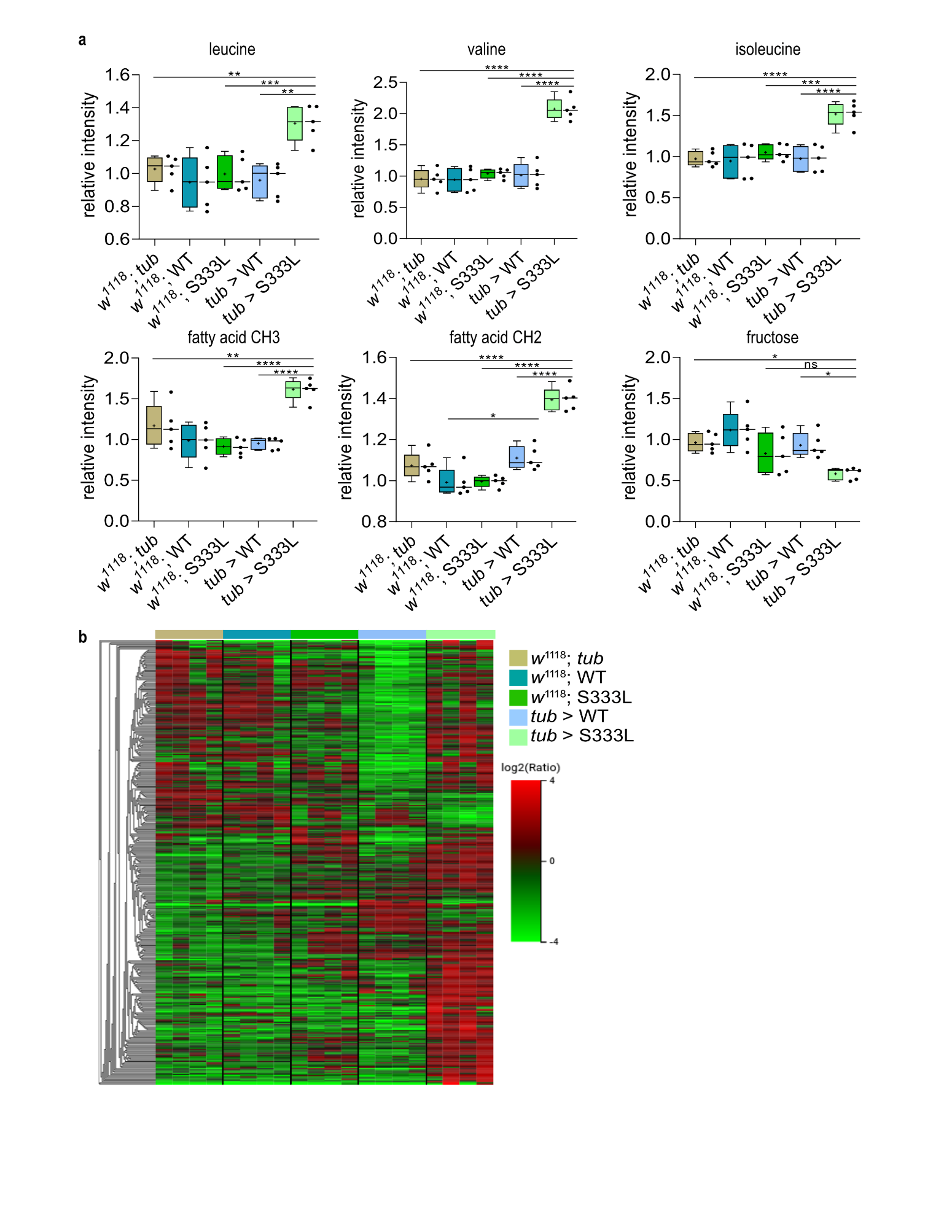


**Supplemental figure 4:** Metabolomic and proteomic analysis of *tub* > CG8111p.S333L animals reveal impaired lipid metabolism

**a:** Fatty acids as well as BCAAs were increased in the mutant compared to the controls. Fructose level was decreased in mutant larvae. **b:** Heat map of proteins with altered regulation obtained from proteome analysis. Data shown as box and wiskers. The median is indicated by the center line of the boxplot; upper and lower bounds indicate 75^th^ and 25^th^ percentiles, respectively; wiskers indicate the minimun and maximum. The average is shown by +. Statistical test: One-way ANOVA test, followed by Dunnett’s Multiple Comparison Test. * = p≤0.05, ** = p≤0.01, *** = p≤0.001, **** = p<0.0001


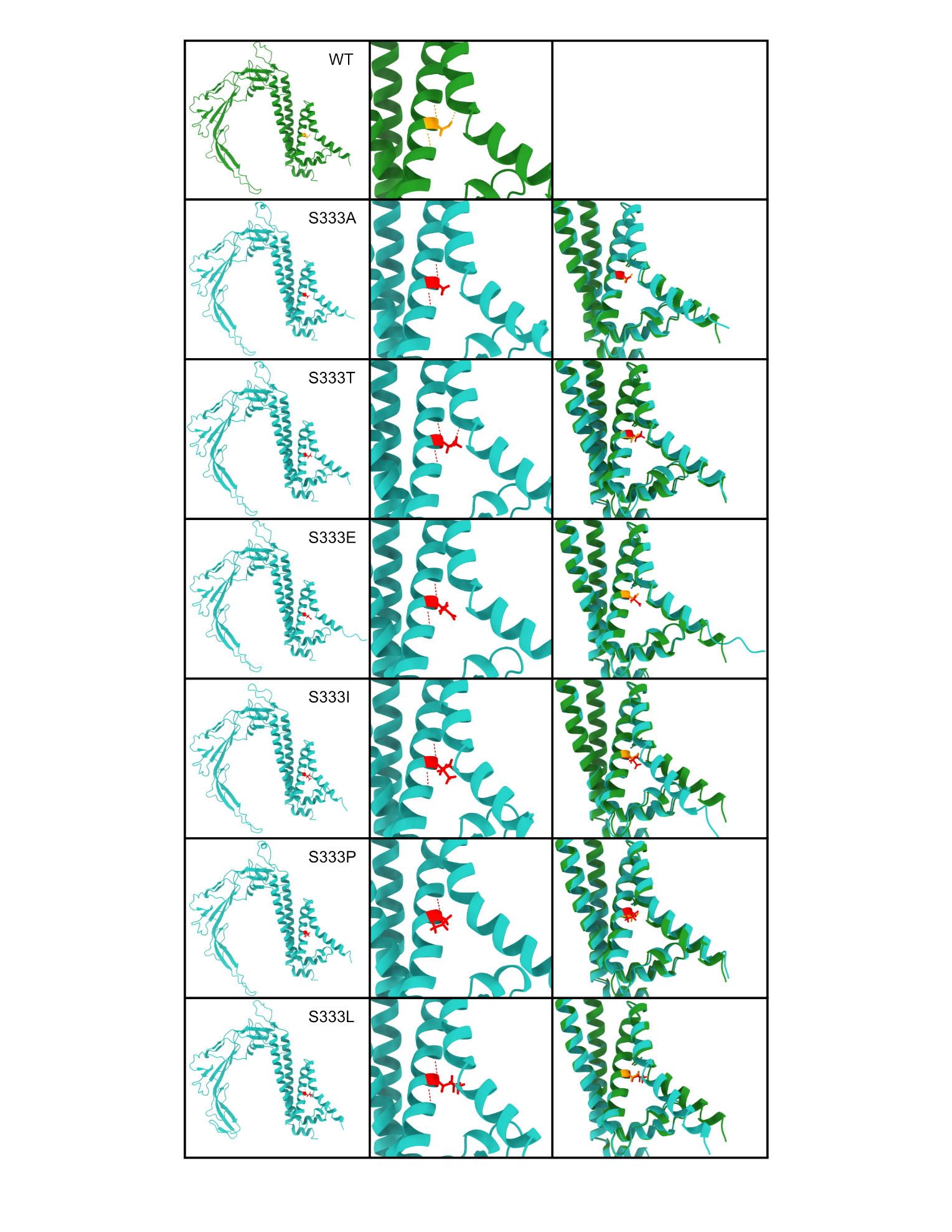


**Supplemental figure 5:** AlphaFold predictions of serine-333 variants

Serine-333 is colored in orange. Serine-333 variants are colored in red. Dotted lines represent hydrogen bonds.

**Supplemental table 1:** Upregulated proteins involved in lipid, fatty acid or BCAA metabolic pathways in CG8111p.S333L overexpressing larvae (#1-12 correspond to the heatmap of Figure 5c**)**

| **Gene** | **Protein** | **Protein class** | **Involved pathways**  **(among others)** |
| --- | --- | --- | --- |
|  |  |  |  |
| mag (1)  (Q9VPE9) | Lipase | Lipase | Lipid metabolic process |
| AcCoAS (2) (Q59E09) | Acetyl-coenzyme A synthetase | Ligase | Fatty acid metabolic process,  Lipid metabolic process |
| CG15093  (A0A0B4JCU8) | 3-hydroxyisobutyrate dehydrogenase | Dehydrogenase | BCAA metabolic process |
| EG:BACR7A4.14  (Q9U1L2) | EG:BACR7A4.14 protein |  | Fatty acid metabolic process,  Lipid metabolic process |
| CG5162 (3) (Q9VX69) | FI01450p | Lipase | Lipid metabolic process |
| CDase (4)  (A4V3N7) | Neutral ceramidase |  | Fatty acid metabolic process,  Lipid metabolic process |
| Acox57D-p (5) (Q9W2G9) | Acyl-coenzyme A oxidase | Oxidoreductase | Fatty acid metabolic process,  Lipid metabolic process |
| CG9512 (6) (Q9VY05) | GH117628p | Oxidoreductase | Lipid metabolic process |
| CG6178 (7) (Q9VCC6) | GM05240p | Ligase | Fatty acid metabolic process,  Lipid metabolic process |
| CG15533 (8) (Q9VA78) | Sphingomyelin phosphodiesterase | Phosphodiesterase | Lipid metabolic process |
| Agpat2 (9)  (Q7KTI0) | 1-acyl-sn-glycerol-3-phosphate acyltransferase | Acyltransferase | Lipid metabolic process |
| CG8199 (10)  (Q9VHB8) | 2-oxoisovalerate dehydrogenase subunit α | Dehydrogenase | BCAA metabolic process |
| Had1 (11)  (Q9VXI1) | β-hydroxy acid dehydrogenase 1, isoform A | Dehydrogenase | Fatty acid metabolic process,  Lipid metabolic process |
| Fatp1 (12)  (E1JHE4) | Fatty acid transport protein 1, isoform F | Secondary carrier transporter | Fatty acid metabolic process,  Lipid metabolic process |

**Movie S1.**

Type or paste caption here.
